# Cloning and characterization of two chlorophyll A/B binding protein genes and analysis of their gene family in *Camellia sinensis*

**DOI:** 10.1038/s41598-020-61317-3

**Published:** 2020-03-12

**Authors:** Xian-Wen Li, Yu-Lin Zhu, Chu-Yan Chen, Zhi-Juan Geng, Xiang-Yong Li, Ting-Ting Ye, Xiao-Nan Mao, Fang Du

**Affiliations:** 1 Nanfang College of Sun Yat-sen University, Guangzhou, 510970 China; 2 College of Life Science, Xinyang Normal University, Xinyang, 464000 China

**Keywords:** Transcriptomics, Photosystem II, Plant molecular biology, Plant stress responses

## Abstract

In this study, two chlorophyll A/B binding protein (CAB) genes (*CsCP1* and *CsCP2*) in tea plant were cloned. The proteins encoded by these genes belong to the external or internal antenna proteins of PS II, respectively. They may be the targets of physiological regulation for tea leaf cell PS II because they all contain multiple functional domains and modifiable sites. The CAB gene family in the tea genome consists of 25 homologous genes. We measured the expression patterns of ten genes in the *CsCP1* and *CsCP2* subfamily under six different stresses. *CsCP1* expression was inhibited in response to 6 kinds of stress; *CsCP2* expression was slightly upregulated only after cold stress and ABA treatment. However, the expression levels of CSA016997 and CSA030476 were upregulated significantly in the six stresses. The results suggested that the 10 CAB genes may have different functions in tea leaves. Moreover, changes in the expression of the 10 genes under stress appear to be related to ABA- and MeJA-dependent signalling pathways, and their responses to MeJA treatment is faster than those to ABA. In addition, we introduced our experiences for cloning the genes in the context of complex genomes.

## Introduction

Chloroplasts are organelle involved in photosynthesis in plants and an important infrastructure to sustain the earth’s ecosystem. The light-harvesting complex (LHCs) consists of proteins and photosynthetic pigments and is an important functional components in chloroplasts. The main components of LHCs are CABs in higher plants. CABs are involved in light uptake, transmit the energy to the reaction centre of two photosystems (PS I and PS II), and adjust the distribution of the excitation energy between them and maintain the structure of thylakoid membrane^1–3^. CAB genes are exclusively encoded in the nucleus genome in higher plants, and are classified into 10 gene families based upon their nucleotide sequence homology. Four CAB gene families including LHCa1, LHCa2, LHCa3 and LHCa4, are associated with PS I, and the 6 CAB families related to PS II contain 3 major LHC II families (LHCb1, LHCb2 and LHCb3) and 3 minor LHC II families (LHCb4, LHCb5 and LHCb6, which encoded CP29, CP26 and CP24, respectively)^4–8^. Although the two photosystems in the thylakoid sub-domains of higher plants are comprised of different antenna proteins, the CAB proteins in PS II have attracted considerable attention gevin their complex physiological functions^4,9^. High homology exists between the major LHCs of PS II, which form homo- or heterotrimers to perform their functions. Compared with the major LHCII family proteins, the minor LHCII family proteins are integrated as monomer into the interior of PS II to absorb and deliver energy to the reaction centre^3,10,11^. CAB genes in some plants (including herbaceous and woody plant, such as *Glycine max* and Pyrus x bretschneideri) have been cloned. CAB gene expression is affected by light intensity, low temperature, high salinity, drought and disease^11–14^.

Evergreen woody plants in subtropical to temperate regions, such as tea plant (*C. sinensis* L.), have to adapt their photosynthetic capacity to the change greatly in light intensity and temperature during different seasons. Study on the genes encoding chloroplast proteins in evergreen woody plants and their expression patterns in various circumstances are important to understand the photosynthesis-mechanism changes in different seasons. There are some different mutant phenotypic cultivars related to the color of young leaves in *C. sinensis*, such as ‘Zijuan’ and ‘Baijiguan’. ‘Zijuan’ has a striking purple phenotype in young foliage and stems given an abnormal pattern of anthocyanin accumulation; ‘Baijiguan’ exhibits a yellow leaf phenotype, reduced chlorophyll (Chl) content, and aberrant chloroplast structures under high light intensity. Although Sun *et al*.^15^ and Wu *et al*.^16^ compared separately the transcriptome changes in ‘Zijuan’ or ‘Baijiguan’ with other tea cultivars’ during different light and temperature treatments, the relationship between tea phenotypes of the purple or yellow leaves and CAB genes were not mentioned in the two papers because the CAB genes were not cloned. Cheng *et al*.^17^ revealed that suppression of the P700-chlorophyll-a protein complex and LHCs might explain the albinism. Ma *et al*.^18^ found that LHCb in the Anji Baicha cultivar was suppressed during the albino stage, but increased dramatically when the shoots turned green. A proteomic analysis of tea leaves showed that CAB proteins were upregulated during drought stress^19^. However, little is known about the expression patterns of CAB genes and their gene families in *C. sinensis*. Here, two CAB genes in tea plants were cloned, and their characteristics, phylogenesis and gene family composition were analysed. This study can provide some information to further explain the functional characteristics and photosynthesis mechanism of CAB genes in tea plants.

## Results

### Cloning and characterization of two genes encoding the CAB of PSII in *C. sinensis*

In the analysis of wound induced transcriptome data (GEFQ00000000) from tea plants, a significantly downregulated CAB protein gene (comp56954_c0_seq1) was identified^20^. Then, we cloned the gene by RACE, and identified another homologous sequence in this process. Two genes encoding CAB proteins in tea plant were named *CsCP1* and *CsCP2* (KY709676 and KY709677) according to their molecular weights. The identity region is 943/949 (99%) between *CsCP1* and comp59744_c0_seq1, a transcript from the wound-induced transcriptome in tea plant (GEFQ00000000), and no gap is observed. The different segments include shorted 15-bp and 10-bp nonsimilarity regions at the 5’ end and the loss of 210 bp at the 3′ end. The similarity region identity between *CsCP1* and CSA004532 (972 bp), which was a gene from the *C. sinensis* genome database (http://www.plantkingdomgdb.com/tea_tree/), is 703/762 (92%), and a gap (30 bp) is noted. *CsCP1* has an additional 30-bp fragment (yellow region). In addition, *CsCP1* is 62 bp longer than CSA004532 at the 5′ end and 29 bp at the 3′ end (blue zone). Moreover, 99% (559/560) identity is noted between *CsCP2* and comp56954_c0_seq1 (977 bp), but *CsCP2* is 270 bp longer at the 5′ end and 417 bp shorter at the 3′ end. The sequence consistency is also 99% (793/798) between *CsCP2* and CSA019572. However, *CsCP2* is 14 bp longer at the 5 ‘end 18 bp longer at the 3′ end compared with CSA019572 (Fig. S1, Table S1).

The nucleotide sequences near *CsCP1* and *CsCP2* translation initiation codon (ATG) were all conformed to the Kozak rule (A/GNNAUGG)^21^ and contained the common TCAC/T sequences of the *CAB* family^22^. The ORF of the *CsCP1* gene is 873 bp in length and encodes a 290-aa protein (30.96 kD, pI 6.33). Moreover, 33.10% a-helix, 37.59% random coil and 19.66% extended strand in the secondary structure of CsCP1 was predicted using SOPMA^23^. Using SMART analysis^24^, a typical CAB domain was composed of the 91- to 254-aa segment (164 aa in length) of the CsCP1 protein, which includes the binding sites for 4 chlorophyll-a, 3 chlorophyll-b and one 1,2-dipalmitoyl-phosphatidyl-glycerole. CsCP1 protein contains a SH3 (Src Homology-3) domain (146 to 207 aa) and two internal repeats in the 105 to 140 and 216 to 251 aa sections (the identity between two internal repeats is 44%) (Table S2). The ORF of *CsCP2* gene is 798 bp in length, and encodes a 265-aa protein (28.65 kD, pI 5.97). The CsCP2 secondary structure contains 38.11% a-helix, 33.21% random coil and 17.36% extended strand. A typical CAB domain is also present in the 65 to 232 aa segment (168 aa in length) of CsCP2 protein, which includes the binding sites of 5 chlorophyll-a and one 1,2-dipalmitoyl-phosphatidyl-glycerole. CsCP2 protein contained a Rho domain (Rho subfamily of Ras-like small GTPases, 67 to 135 aa sections) and two internal repeats in the 73 to 122 and 189 to 237 aa sections (the identity between two internal repeats is 34%) (Table S2). Five predicted N-myristoylation sites are located in the 28–33, 32–37, 108–113, 122–127 and 178–183 aa segments of CsCP2 protein, and the 3 phosphorylation sites of protein kinase C are located in the threonines of 36–38, 40–42 and 234–236 aa segments of CsCP2. The nucleotide sequence alignment between *CsCP1* and *CsCP2* revealed two high similarity segments in a 222-bp 3′-terminal segment (the identity was 67%) and a 193-bp 5′-terminal segment (the identity was 70%) in the two genes, but the similarity of the other parts in their sequences was very low. The N-terminal regions of both proteins had a chloroplast transit peptide for chloroplast localization.

**Figure 1:**
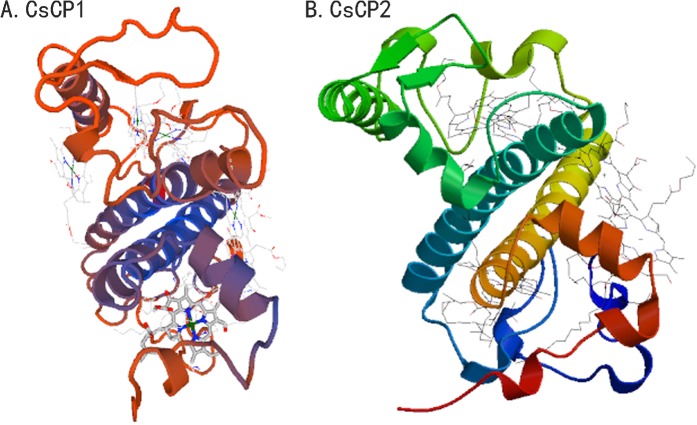
The predicted tertiary structure of CsCP1 and CsCP2 proteins.

CsCP1 protein is the homologous protein of LHCb5 (KEGG orthology term K08916) as determined by KEGG pathway analysis, which suggested that it was the internal antenna protein of PS II. CsCP2 belongs to the external antenna protein of PS II because it is homologous to LHCb2 protein (KEGG orthology term K08913). Through SWISS-MODEL and Phyre2 analysis, the 214-aa residues of CsCP1 (74% of CsCP1 sequence) were modelled to cryoEM structure of spinach PSII-LHCII by the single highest scoring template with 100.0% confidence, and the 218-aa residues of CsCP2 (82% of CsCP2 sequence) were modelled to a Chlorophyll A/B binding protein. CsCP1 and CsCP2 had similar tertiary structures that all contained three a-helices that were integrated in the thylakoid membrane (Fig. 1). However, they have difference in binding pigment molecule types and numbers in other regions of the proteins except the CAB domain.

### The CAB protein family constituents in *C. sinensis* and the phylogenetic relationship

**Figure 2:**
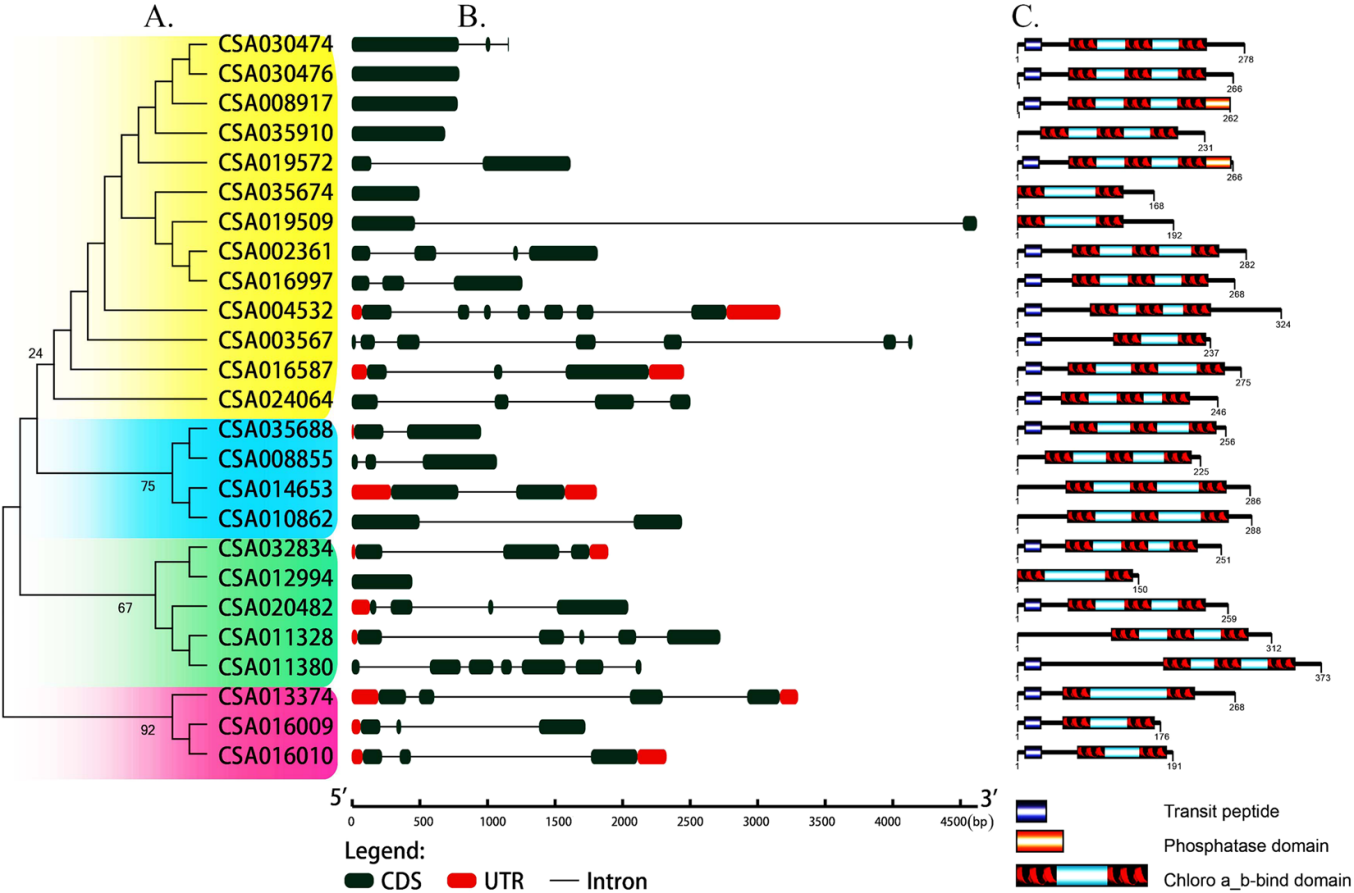
CAB gene family members and the structures of their encoding proteins in *C. sinensis*.

To identify protein homologous to CsCP1 and CsCP2 in tea plant, the genome database^25^ was searched by using the two CsCP1 and CsCP2 sequences as queries via Blastp with a threshold of E-value < 1e-5. A total of 25 homologous protein sequences were obtained after assessing for the presence of the Chloroa/b-bind domain in all obtained sequences. Phylogenetic analysis of the 25 sequences (MEGA6.0) indicated that they could be divided into 4 subfamilies, *CsCP1* (CSA004532) and *CsCP2* (CSA019572) were classified in the same subfamily, which contained 13 members (Fig. 2A, Table S3 and S4). There was only one amino acid difference between CsCP2 and the protein encoded by CSA019572.1 which was most similar sequence of *CsCP2*. However, there were 2 gaps (10 and 43 aa) between CsCP1 and its most similar sequence, the protein encoded by CSA004532.1, although the other sections were almost identical (Table S2).

Given the restriction of the tea tree genome assembly at present^25,26^, the genes encoding the 25 homologous proteins of CsCP1/ CsCP2 can not be localized to specific chromosomes in *C. sinensis*. However, the gene features of the abovementioned 25 genes are displayed by using GSDS2.0 (Gene Structure Display Server http://gsds.cbi.pku.edu.cn/) according to *C. sinensis* genome annotation^25^. Some of the genes did not contain introns, and others contained 1–6 introns (Fig. 2B). All of the 25 proteins encoded by the *CAB* gene family in tea plant contain a CAB domain, which consist of 2 or 3 α-helices. Seventeen of the genes have a chloroplast transport peptide in the N-terminus in addition to 8 additional sequences (Fig. 2C, Table S2).

**Figure 3:**
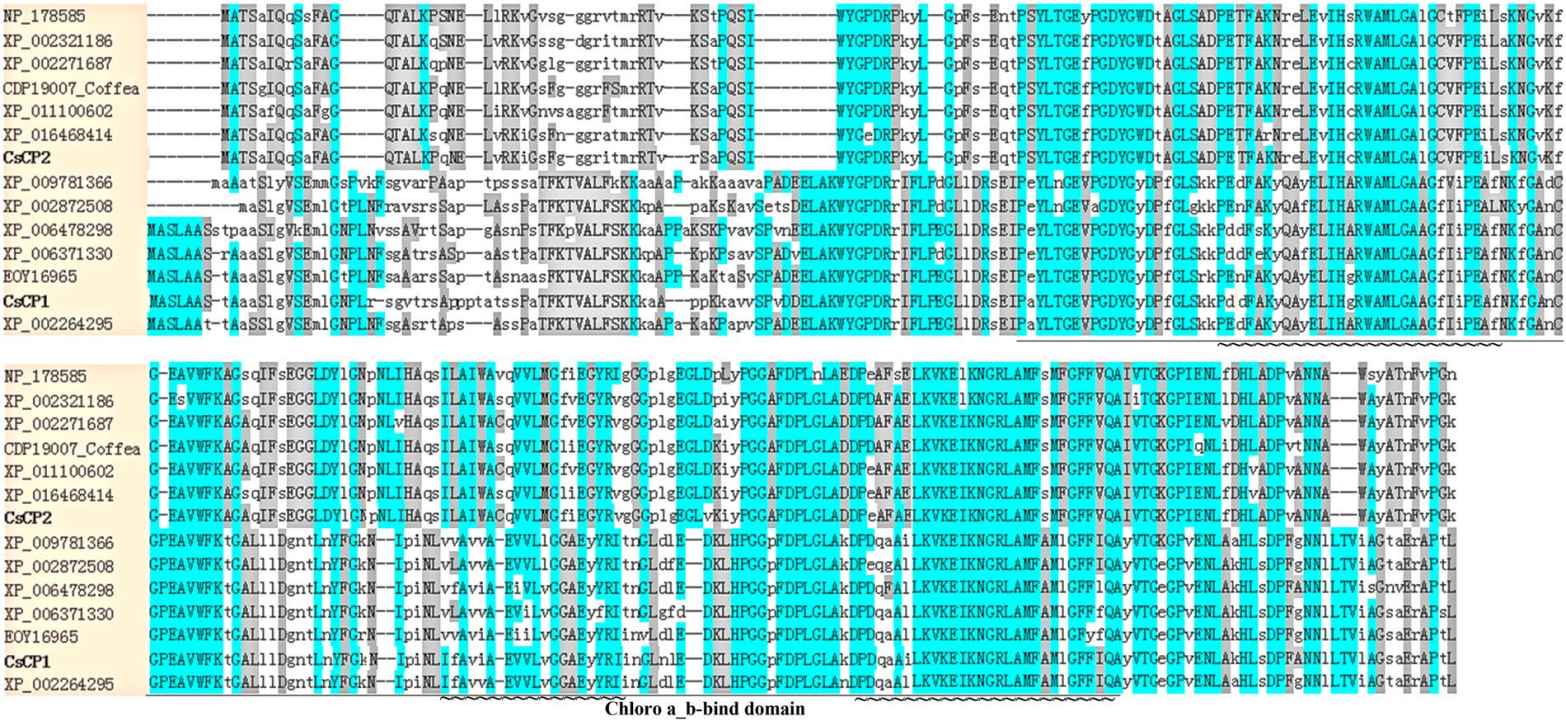
Sequence alignment between CsCP1 and CsCP2 with homologous proteins in other species.

To understand their evolutionary trajectories in the plant kingdom, proteins homologous to CsCP1 and CsCP2 were searched by using BlastP in NCBI. Greater than 100 homologous sequences of CsCP1 were obtained, including XP_012072637.1 (*Jatropha curcas*), XP_006478298.1 (*Citrus sinensis*), XP_002264295.1 (*Vitis vinifera*) and XP_010063732.1 (*Eucalyptus grandis*), all of which belonged to the chlorophyll A/B binding protein CP26. The sequence identities between CsCP1 and these proteins were greater than 76%, and the total score was greater than 440. Moreover, the sequence between CsCP1 protein and a hypothetical protein in *Paenibacillus* sp. IHB B 3415, WP_039310936, was completely identical. Greater than 100 CsCP2 homologous proteins were logged in NCBI, including XP_002531690.1 (a chlorophyll a-b binding protein 151, chloroplastic in *Ricinus communis*), XP_009802594.1 (a chlorophyll a-b binding protein 36 in *Nicotiana sylvestris*), XP_002271687.1 (chlorophyll a-b binding protein 151 in *Vitis vinifera*), XP_021664574.1 (a chlorophyll a-b binding protein 151 in *Hevea brasiliensis*). The sequence identities between CsCP2 and its homologous proteins were greater than 90%, and the total score were greater than 500. Sequence alignment was performed using 7 proteins selected from CsCP1 or CsCP2 homologous proteins, separately. The results indicated that most of the sequences of the two sets of proteins were highly similar, with the exception of their N-terminus regions and the regions near the second helix of the chlorophyll A/B binding domain (Fig. 3).

### The expression profiles of the CAB genes in *C. sinensis*

**Figure 4:**
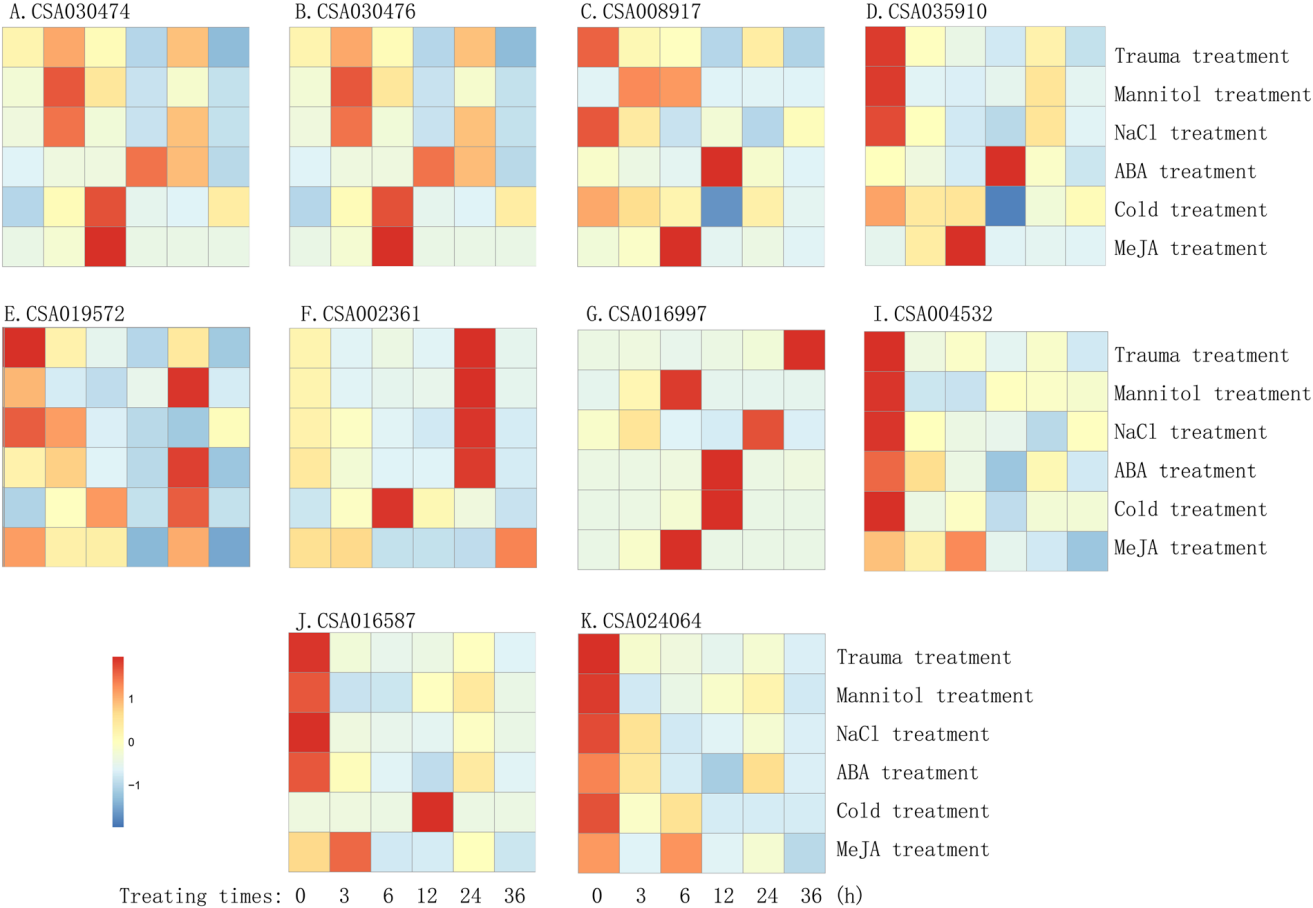
Expression changes of 10 CAB genes in tea plant during 6 different treatments.

To understand the expression patterns of the CAB gene family in tea plant, the expressions of 25 *CAB* genes after wounding was analysed firstly using the transcriptome data (GEFQ00000000)^20^. The result shows that the expression of almost all of the genes of the subfamily of *CsCP1* and *CsCP2* was significantly altered. The other three subfamily genes exhibited low expression with minimal changes (Fig. S2). Of the 13 genes in the *CsCP1*/*CsCP2* subfamily, 3 genes (4th, 6th and 7th in Fig. 2: CSA035910, CSA035674 and CSA019509) lacked the coding region for the chloroplast transporter peptide. The expression levels of 6th and 7th genes were very low after wounding. The 11th gene in Fig. 2, CSA003567, is similar to 6th and 7th genes. The expression of these genes is very low, and their encoded proteins have only two transmembrane helix domains (Fig. 2; Table S2). Therefore, the three genes were not further analysed, and the expression patterns of other ten genes of the *CsCP1* and *CsCP2* subfamily in response to different stress conditions were assessed by qRT-PCR. The primers were designed in the differential region according to the sequence alignment of the CAB gene family in tea plants, and no nonspecific amplification occurred as verified by PCR (Fig. S3; Table S5). The main results are as follows: 1. During the 6 different stress treatments, the expression levels of only two of the 10 genes, CSA030476 and CSA016997, were upregulated transitorily greater than 1.5-fold compared with expression levels at 0 h′; Only CSA002361 was all upregulated momently in five stress conditions; CSA030474 and CSA008917 were upregulated in response to the three stressors. Three genes (CSA035910, CSA019572 and CSA016587) were upregulated by greater than 1.5-fold in two stress treatments. Of the upregulated 8 genes, 5 expressions were upregulated for longer than 3 h. In the other words, the time spans of the upregulated expressions were more than 2 serial sampling points, and the longest upregulated time was greater than 20 h (from 3 to 24 h sampling points). Six genes were upregulated transitorily by more than 4-fold in treatments, and the highest expression change was an 18011-fold increase compare with no treatment. However, two genes (CSA004532 and CSA024064) were all downregulated in response to six stressors. In addition, the expression levels of the abovementioned upregulated 8 genes were downregulated at the other sampling points, and their transcripts could not be detected at the most severe periods of downregulation (the expression level was 0). Some genes with closer genetic relationships to each other exhibit more similar expression patterns, for example, the expression patterns of CSA030474 and CSA030476, as well as CSA004532 and CSA024064 were similar. The expression patterns of CSA002361 and CSA016997 were also similar. However, the expression patterns of other genes were significantly different (Fig. 4; Table S6). 2. After mechanical injury, the expression levels of four (CSA030474, CSA030476, CSA002361 and CSA016997) of the 10 genes fluctuated between 0.02- and 12.82-fold that of the control expression levels. This result is different from that of the wound-induced transcriptome (GEFQ00000000), in which all of the genes were downregulated (Fig. S2 and S4A; Table S6). The expression levels of abovementioned 4 genes fluctuated between 0.01- and 4.2-fold that of the controls after NaCl treatment (Fig. S4D; Table S6). During the mannitol treatment, cold treatment, Methyl jasmonate (MeJA) treatment or abscisic acid (ABA) treatment, the expression levels of 4, 5, 5 and 7 genes were briefly upregulated by greater than 1.5-fold, respectively (Fig. S4C,B,F and E; Table S6). 3. The expression of *CsCP1* gene was inhibited under the abovementioned six stress conditions, especially mannitol or NaCl treatment, and levels were reduced to less than 1/10 of the baseline value at some sampling points. *CsCP2* gene expression was downregulated or not significantly changed in response to the four stress conditions, but was briefly upregulated to greater than 1.5-fold that of the control under cold and ABA treatment. Obviously, the expression pattern of *CsCP1* is different from that of *CsCP2*. Moreover, it were also significantly different that the expression patterns of the genes between *CsCP1* and CSA030476 or CSA016997, or between *CsCP2* and CSA035910 or CSA008917, the coding proteins of which were with the highest similarity in amino acid sequences in the CAB gene family of *C. sinensis* (Table S7).

## Discussion

Chloroplast has been always a research hotspot in the field of life sciences because it is a photosynthesis organelle in plant cells. However, it is also one of the most sensitive structures to multiple environmental stresses (e.g. cold, drought and wound) and plays a very important in plant stress response. Transcriptome analysis of chloroplast developmental defect mutants in *Hordeum vulgare* showed that approximately 67% of the cold regulatory genes were dependent on the organelle, and only 11% were chloroplast independent. The adaptation of plants to cold environment is achieved by changing the expression levels of genes that are closely related to the signal transduction from the chloroplast to cell nucleus^27^. Tea plants, as evergreen plant, are subjected to heat from strong light in summer and cold with excess light in winter, so their leaves represent an ideal material for studying photosynthesis mechanisms during cold/hot temperatures with strong light stress. To breed new variety of tea plants with early buds and freeze-resistant properties, our team mainly focused on the low temperature response in tea leaf and the chloroplast development^28,29^. Tea plants are also often subjected to picking and pruning injury given the economic significance of tea leaves. In this study, *CsCP1* and *CsCP2* genes were cloned according to wound-induced transcriptome data in tea plants^20^. It would be not easy to clone the corresponding genes using a RACE strategy based on the assembly sequences from a transcriptome due to the following reasons: 1. Some of the assembly sequences from the transcriptome data are not sufficiently accurate, especially given the absence of reference genome data or the lower quality of reference genome data^25^. 2. If a RACE strategy is used to clone a target gene of a polygenic family in a species, it is often interfered by the high similarity of nucleotide sequences among its members. 3. Multiple intron genes often produce some different cDNA sequences via selective splicing. Therefore, we experienced multiple failures in cloning the *CsCP2* gene at the beginning of the study due to the abovementioned possible reasons. The two products, *CsCP1* and *CsCP2*, were obtained though some optimization measures, such as analysing the gene family and re-designing the primers. It can be observed that the selection of primer locations is critical when we clone a gene according to the assembly sequence from a transcriptome or genome using RACE method, The primer would be avoided designing in the assembly regions where errors may occur and in the conserved sequence regions of the gene family, which is more important for qRT-PCR primer designs. There are two inferences that might be drawn from the abovementioned analysis and our results. 1. The sequence differences between *CsCP1*/*CsCP2* and its corresponding assembled sequence based on the transcriptome or genome may result from the sequence is not assembled accurately (Fig. S1, Table S1). 2. The expression differences between *CsCP1/CsCP2* and its corresponding assembled sequence may result also from the sequence is not assembled accurately (Figs. S2 and S4A, Table S6). Moreover, CsCP1 was completely identical to WP_039310936, a hypothetical protein from *Paenibacillus sp*. IHB B 3415. This finding represents an interesting problem given that a chlorophyll binding protein in tea plant has the same sequence as a protein in bacterial cells.

Plants are subjected to a variety of environmental stresses in their life cycles, and these stressors will directly or indirectly affect their photosynthesis processes. If CAB proteins play a rigid role, excess of light energy will be received because CAB proteins play an important role in the process of receiving and transmitting light energy. This situation will cause light damage to the photosynthetic apparatus of the chloroplast and even the entire mesophyll cell. Therefore, under environmental stress, CAB may represent a pivotal regulatory site of photosynthesis. Cells can regulate CAB activity at many levels, such as gene and protein levels. Structural analysis shows that CsCP1 protein contains a SH3 domain (Src Homology-3), and CsCP2 protein contains a Rho domain (Rho subfamily of Ras-like small GTPases), 3 protein kinase C phosphorylation sites and a phosphatase domain in its C-terminal (Table S2). These results suggest that the two proteins, especially CsCP2, can be used as targets for physiological regulation in the PS II of tea plant cells, and are affected by a variety of regulatory proteins. Furthermore, it suggests that phosphorylation/ dephosphorylation and GTP may be the main forms of the above regulation. The regulation of CAB protein levels enables chloroplasts to respond flexibly and rapidly to environmental stresses. In contrast, regulation at the genetic level has a lasting effect, although it is a delayed response. The CAB gene family in tea plant genome consisted of 25 members. There are 15, 9, and 15 CAB genes in the genome of *Arabidopsis thaliana*, rice or *Populus*, respectively^30,31^. Tea has more CAB genes than herbs (e.g., *A. thaliana* and rice) and deciduous woody plant (e.g., *Populus*), which may be related to the need for the leaves of evergreen woody plants to respond to more environmental stresses. In this study, the expression patterns of 10 genes in the *CsCP1* and *CsCP2* subfamily under six different stress conditions were detected by qRT-PCR to understand the role of CAB genes in tea plant. The expression levels of 8 genes were upregulated briefly by greater than 1.5-fold. The upregulation of CSA016997 and CSA030476 was the most significant among the 8 genes, and the upregulation was transitory or fluctuated in response to the six stresses. The upregulation of CSA016997 expression was the most rapid and significant for cold, mannitol and MeJA treatment. The response persisted from 3 to 6 or 12 h, and the expression level was upregulated by greater than 210-, 18011- or 2515-fold, respectively. The upregulation of CSA030476 was also very quick and significant in response to MeJA treatment, which was upregulated by 325- and 3516-fold compared with control at 3 to 6 h, respectively. The CSA002361 gene, which was the third gene most regulated, was upregulated under 5 stress treatments. However, regarding the 2 genes cloned in this study, *CsCP2* gene expression was slightly upregulated only after cold stress and ABA treatment, while the expression level was downregulated at the other time points and the other four treatments. *CsCP1* gene expression was inhibited in responsed to 6 types of stress (Fig. 4; Table S6). These findings may be directly related to the fact that CsCP1 and CsCP2 proteins are components of the pigment protein complex of the light-trapping antenna. Under stress conditions, reducing the synthesis of the light-trapping antenna proteins prevents the structure from receiving excessive light energy and thus reduces or avoids photooxidation^11,32^. According to this logic, the proteins encoded by the CSA016997, CSA030476 and CSA002361 genes, which were upregulated in response to stress conditions, should not be used to receive and transmit light energy, but rather to dissipate excess light energy or participate in photoprotection.

ABA and MeJA are all important phytohormones that modulate the plant respond to stressful conditions^33–35^. To explore the relationship between the expression regulation of CAB genes in tea plants and MeJA or ABA hormone signal transduction pathways under stress conditions, we analysed the expression changes in the CAB genes in tea leaves exposed to exogenous ABA or MeJA hormones. The results revealed that the expression levels of 7 and 5 genes were upregulated greater than 1.5-fold in response to ABA and MeJA treatments, respectively, compared with control. However, the expression of other genes was downregulated. These results suggest that the expression changes of the CAB genes in tea plant under stresses are related to ABA- and MeJA-dependent signalling pathways^32,36,37^. The upregulation of gene expressions were induced by MeJA occurs mostly at 3 or 6 h. The upregulation of gene expressions induced by ABA occurs mostly at 12 to 24 h, but a slight reduction occurred before the upregulation, suggesting that the responses of the CAB genes in the tea leaves to the MeJA is more rapid compared with that of ABA. However, the expression changes of analysed 10 CAB genes in tea plants in response to wound, cold, dehydration and high salt stress occurred from 3 h to 36 h. These expression changes and the upregulation timelines of some genes induced by the hormone and other stress treatments are closely related to each other, such as CSA016997. In contrast, the expressions of other genes exhibits minimal correlation with ABA and MeJA, such as CSA004532 and CSA024064 (Fig. S4; Table S6). These findings indicate: 1. the expression regulations of *CABs* in plant in response to stress may involve a variety of signal pathways, not just MeJA- and ABA-dependent pathway; 2. The functions of analysed 10 CAB genes in the *CsCP1* and *CsCP2* subfamily in tea plants may be more or less different, because their expression regulation in tea leaves in response to stresses are different.

In this study, two *CAB* genes in the tea plant were cloned, their gene family was analysed, and the expression patterns of ten genes in the *CsCP1* and *CsCP2* subfamily under six different stress conditions were measured by qRT-PCR. Our results provide some valuable data for further studies on photosynthesis and the function of *CAB* genes in tea plants.

## Methods

### Materials and treatments

Tea seedlings with consistent growth of 2-year-old clones from tea plant (*C. sinensis* L. cv. Xinyangdaye) were transplanted to pots in greenhouse. After 1 month of adaptation, the tea seedlings with consistent growth were moved into the artificial climate chamber (25 ± 1 °C; light/dark cycle, 14/10 h; light intensity, 500 μmol/m2/s; humidity, 75%) for 2 weeks. Then, the seedlings were treated and sampled.

For cold treatment, 10 tea seedlings were transferred to a growth chamber maintained at 2 °C.

For wounding treatment, each leaf of 20 plants was cut on both sides of the vertical midrib^20^.

For ABA/MeJA treatment, 20 plants were sprayed with ABA solution (100 μM)/ MeJA solution (45 µM), while the control plants were sprayed with water. The plants were maintained in the same growth chamber^38,39^.

The experimental materials used for dehydration stress were the branches of tea trees. The branches were washed with tap water immediately after being cut off to remove the wound hormones, immersed in 1/2 MS solution for one week to recover in the artificial climate chamber, and then transferred to mannitol solution (300 mM) for dehydration stress treatment^38^.

Each of the above treated materials were sampled at 0 (control check, CK), 3, 6, 12, 24 h and 36 h after the treatment. All of the first samplings (0 h) was conducted at 9:00 am.

All sampled materials were dipped immediately in liquid nitrogen and stored at a − 80 °C until use for RNA extraction.

### Gene cloning and bioinformatic analysis of CAB sequences

Target genes were cloned from a tea plant cDNA library by RACE (rapid-amplification of cDNA ends) strategies using specific primers (Table S5) according to the previous experimental data of our laboratory. Homology search of CAB proteins was performed using BlastP (http://www.ncbi.nlm.nih.gov/BLAST/). ProtParam (http://www.expasy.org/tools) were used to compute the various physical and chemical parameters of CAB proteins. Protein structure analyses and domain identifications were enforced in SMART (http://smart.embl-heidelberg.de/), NCBI CDD^40^ (http://www.ncbi.nlm.nih.gov/cdd/), MOTIFSCAN (http://prosite.expasy.org/), Pfam^41^ (http://pfam.xfam.org/) and SWISS-MODEL (https://swissmodel.expasy.org/interactive) with default parameters to search for conserved domains or phyre2 (http://www.sbg.bio.ic.ac.uk/phyre2/html/page.cgi?d=index) in normal mode^42^. The transit peptides in proteins were predicted by ChloroP^43^ (http://www.cbs.dtu.dk/services/ChloroP/).

### Analysis of CAB protein family members in *C. sinensis* and phylogenetic relationships

The *C. sinensis* genome database (http://www.plantkingdomgdb.com/tea_tree/ and http://www.yunchakj.com/index. aspx?lanmuid=63&sublanmuid=691&id=1367) was searched to identify the CAB domain-containing proteins using Blastp. Pfam and SMART were used to obtain protein sequences from the Chloroa/b-binding domain (PF00504, cl02879) using the Hidden Markov Model (HMM).

The sequences of CAB domain were used to identify conserved amino acid residues for interaction with co-factors. Multiple sequences alignments and regenerating phylogenetic tree were performed in Phylogeny.fr (http://www.phylogeny.fr/simple_phylogeny.cgi) using the Neighbour-Joining (NJ) method, and bootstrap analysis was conducted using 1000 replicates with the p-distance model.

### Analysis of the expression profiles of the *CsCP1* and *CsCP2* gene family

qRT PCR analysis of *CsCP1* and *CsCP2* expression was performed using the ABI PRISM 7300 Real-Time PCR System (Applied Biosystems) according to the procedures by Wang *et al*.^44^. The qRT PCR primers (Table S2) were designed for differential sequence regions of the ten genes in the CsCAB family (Tables S3 and S4) using DNAClub software. The β-actin gene (HQ420251) of tea plant was used as reference for mRNA expression. Total RNA for qRT PCR was extracted from tea leaves via abovementioned treatments by using *TaKaRa* MiniBEST Plant RNA Extraction Kit (Code No: 9769). cDNA was synthesized from the total RNA (4 μg) using a PrimeScript^TM^ RT Master mix (Perfect Real Time) (*TaKaRa* Code No. RR036A).

## Supplementary information

SUPPLEMENTARY INFO.
