# Supplementary material for: Cloning and characterization of two chlorophyll A/B binding protein genes and analysis of their gene family in Camellia sinensis
Source: Sci Rep. 2020 Mar 12;10:4602. doi: 10.1038/s41598-020-61317-3 (PMC7067855; doi:10.1038/s41598-020-61317-3)
Supplement: Supplementary file 1 — SUPPLEMENTARY INFO. [file 41598_2020_61317_MOESM1_ESM.pdf]

Cloning and characterization of two chlorophyll A/B binding protein genes and analysis of their gene family in *Camellia sinensis*

Xian-Wen Li[1,2*], Yu-Lin Zhu[1], Chu-Yan Chen[1], Zhi-Juan Geng[2], Xiang-Yong Li[2], Ting-Ting Ye[2], Xiao-Nan Mao[2], Fang Du[2]

1. Nanfang College of Sun Yat-sen University, Guangzhou, 510970; 2. College of Life Science, Xinyang Normal University, Xinyang 464000, China.

*Correspondence and requests for materials should be addressed to X.W.L.(xianwenli01@sina.com)

Supplementary Figure S1–S4 and Table S1–S7

1. Supplementary Figure S1–S4

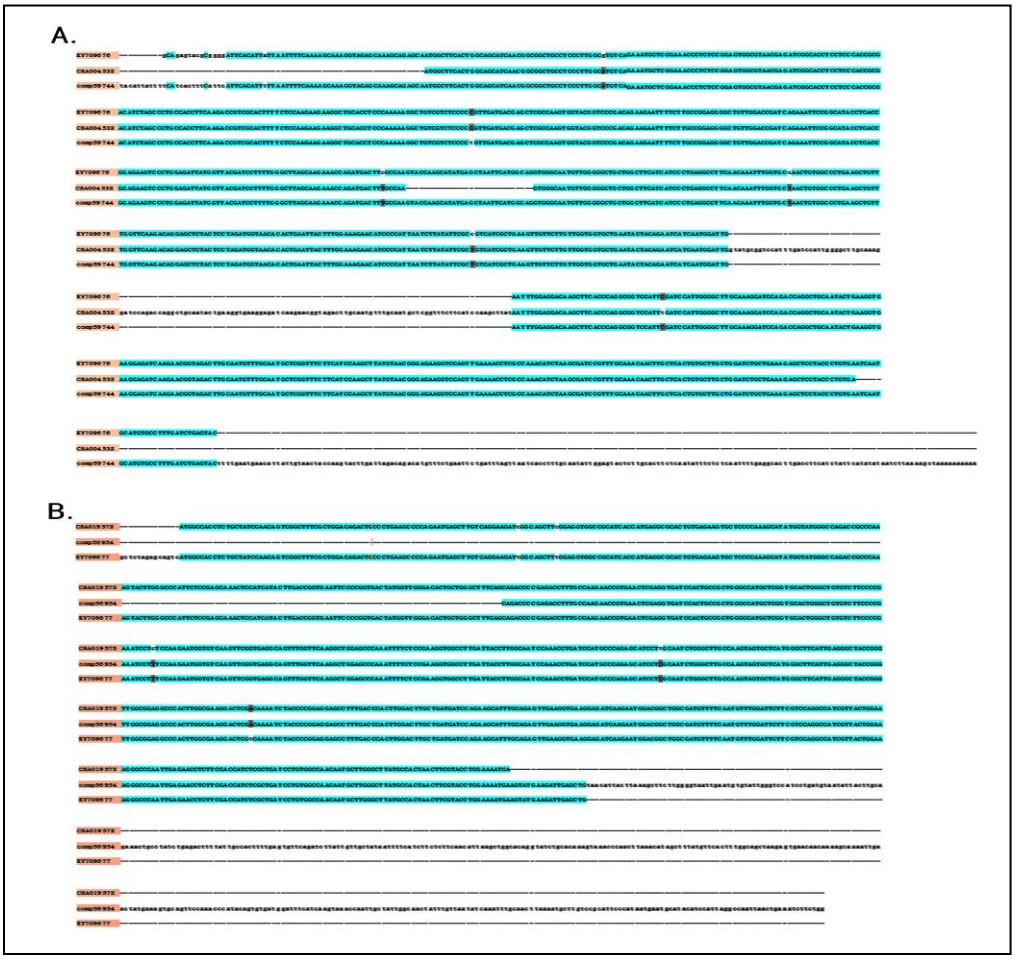

1) Figure S1. The alignment of *CsCP1* or *CsCP2* with corresponding assembly sequences from the tea genome or wound-induced transcriptome.

A.

geneID	sample_0h	sample_3h	sample_6	sample_12	sample_24l
CSA030474	2808.019	1493.192	1858.551	16.23293	1183.024
CSA030476	9779.017	6888.705	11148.2	170.2105	4739.542
CSA008917	17992.22	15516.38	16120.65	1756.076	15367.97
CSA035910	7165.052	5583.499	6450.302	219.8899	2817.451
CSA019572	9933.674	7358.302	7587.571	415.2169	7310.101
CSA035674	12.11154	0.430419	0.727143	0	2.609951
CSA019509	0	0	0	0	0
CSA002361	4817.612	1573.562	1378.657	60.50817	2475.895
CSA016997	159.5478	31.13788	17.33367	0	46.4399
CSA004532	1818.338	1527.41	1653.725	755.8831	1593.36
CSA003567	30.56927	19.89036	23.02499	32.60939	28.93091
CSA016587	5132.047	3196.941	2722.814	715.9075	4510.967
CSA024064	4372.392	2643.653	3986.862	895.937	3127.108
CSA035688	796.954	355.5643	453.1161	35.43377	401.028
CSA008855	2259.42	1241.54	1048.283	146.1004	1340.188
CSA014653	410.1606	200.4732	363.5718	116.9905	239.1617
CSA010862	3164.027	2180.368	3217.486	989.251	2556.845
CSA032834	3431.402	2345.687	3190.032	291.0349	2695.446
CSA012994	927.7938	388.1887	681.7132	182.3219	609.2958
CSA020482	150.252	67.63686	56.40336	85.21569	129.2637
CSA011328	4572.572	2953.866	3400.094	874.9939	4156.478
CSA011380	277.5138	215.2938	295.8016	12.38341	87.551
CSA013374	1170.146	729.4614	633.1982	680.28	1083.935
CSA016009	32.34863	24.76749	0	2.742393	78.83485
CSA016010	283.3229	85.9076	12.00024	11.87337	416.1134

B.

0 h	3 h	6 h	12 h	24 h
1	0.5318	0.6619	0.0058	0.4213
1	0.7044	1.1400	0.0174	0.4847
1	0.8624	0.8960	0.0976	0.8541
1	0.7793	0.9002	0.0307	0.3932
1	0.7407	0.7638	0.0418	0.7359
1	0.0355	0.0600	0.0000	0.2155
1	0.0000	0.0000	0.0000	0.0000
1	0.3266	0.2862	0.0126	0.5139
1	0.1952	0.1086	0.0000	0.2911
1	0.8400	0.9095	0.4157	0.8763
1	0.6507	0.7532	1.0667	0.9464
1	0.6229	0.5306	0.1395	0.8790
1	0.6046	0.9118	0.2049	0.7152
1	0.4462	0.5686	0.0445	0.5032
1	0.5495	0.4640	0.0647	0.5932
1	0.4888	0.8864	0.2852	0.5831
1	0.6891	1.0169	0.3127	0.8081
1	0.6836	0.9297	0.0848	0.7855
1	0.4184	0.7348	0.1965	0.6567
1	0.4502	0.3754	0.5672	0.8603
1	0.6460	0.7436	0.1914	0.9090
1	0.7758	1.0659	0.0446	0.3155
1	0.6234	0.5411	0.5814	0.9263
1	0.7656	0.0000	0.0848	2.4370
1	0.3032	0.0424	0.0419	1.4687

2) Figure S2. The expression changes of CAB gene family members in trauma-induced transcriptome of tea plant. A. RPKM. B. Relative expression (0 h as reference point).

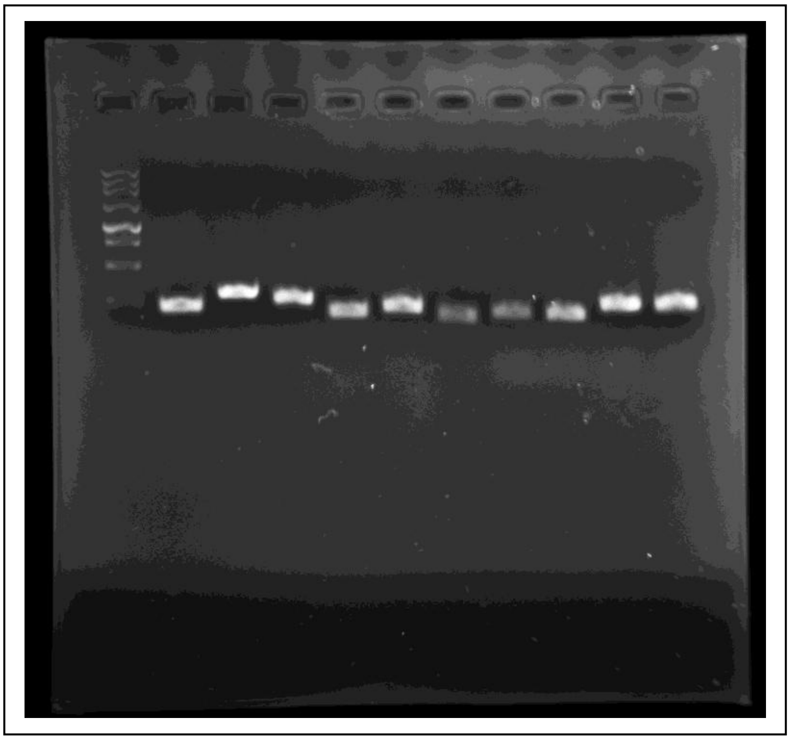

3) Figure S3. PCR tests on the specificity of qRT-PCR primers of 10 CAB genes in tea plants.

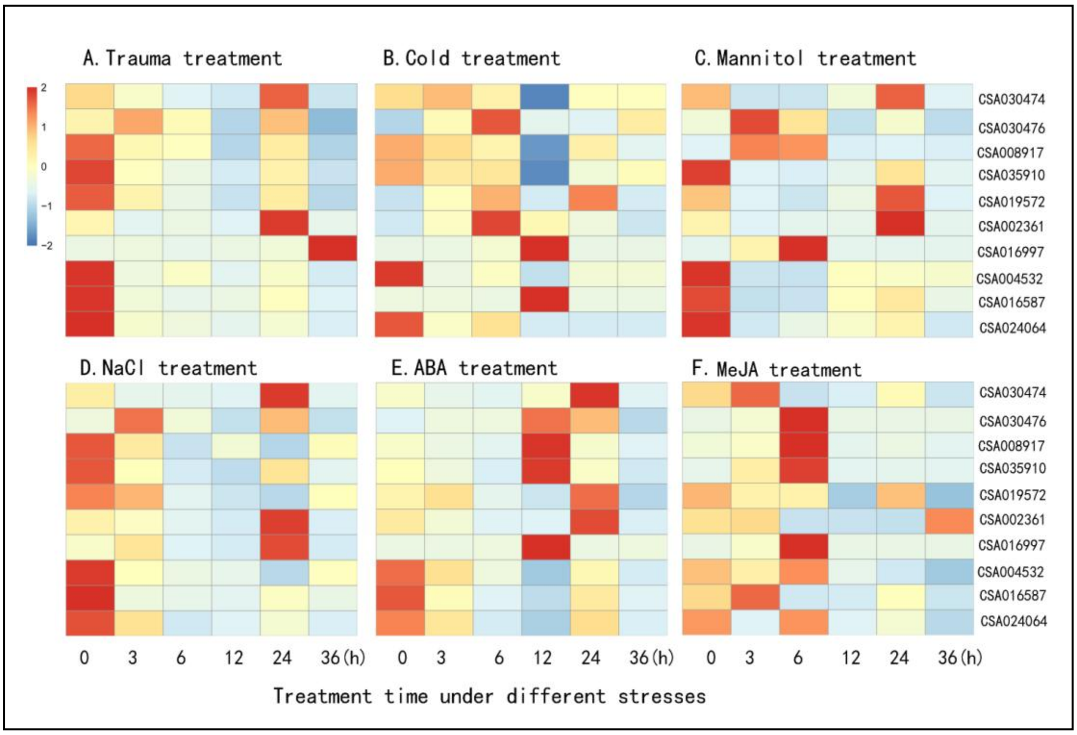

4) Figure S4. The expression patterns of 10 CAB genes in tea plants under 6 different treatments.

2. Supplementary Table S1–S7

1) Table S1. The sequence and alignments of *CsCP1* and *CsCP2* with the corresponding assembly sequences

(1) ***CsCP1*** (KY709676, 964 bp), The identity region is 943/949 (99%) between *CsCP1* and comp59744_c0_seq1, and is no gap; The different segments include shorting 15-bp and 10-bp nonsimilarity at 5' end, and 210 bp less at 3' end. The similarity region identity between *CsCP1* and CSA004532 (972 bp) is 703/762 (92%), of which has a gap (30 bp), *CsCP1* has an additional 30 bp fragment (yellow region). In addition, *CsCP1* is 62 bp longer than CSA004532 at 5 'end and 29 bp at 3' end (blue zone).

① ***CsCP1*** sequence was amplified by RACE and submitted to the Genbank

GCAGAGTACGCGGGGATTCACATTATTAATTTTCAAAAGCAAAGGTAGAGCAAAGCAGAGCAATGG
CTTCACTGGCAGCATCAACGGCGGCTGCCTCCCTTGGCGTGTCAGAAATGCTCGGAAACCCTCTCCG
GAGTGGCGTAACGAGATCGGCACCTCCTCCCACCGCGACATCTAGCCCTGCCACCTTCAAGACCGTC
GCACTTTTCTCCAAGAAGAAGGCTGCACCTCCCAAAAAGGCTGTCGTCTCCCCCGTTGATGACGAGC
TCGCCAAGTGGTACGGTCCCGACAGAAGAATTTTCTTGCCGGAGGGGCTGTTGGACCGATCAGAAAT
TCCGGCATACCTCACCGGAGAAGTCCCTGGAGATTATGGTTACGATCCTTTTGGGCTTAGCAAGAAA
CCAGATGACTTCGCCAAGTACCAAGCATATGAGCTAATTCATGGCAGGTGGGCAATGTTGGGGGCTG
CTGGCTTCATCATCCCTGAGGCCTTCAACAAATTTGGTGCCAACTGTGGCCCTGAAGCTGTTTGGTTC
AAGACAGGAGCTCTACTCCTAGATGGTAACACACTGAATTACTTTGGAAAGAACATCCCCATTAATC

TTATATTCGCCGTCATCGCTGAAGTTGTTCTTGTTGGTGGTGCTGAATACTACAGAATCATCAATGGA
TTGAATTTGGAGGACAAGCTTCACCCAGGCGGTCCATTCGATCCATTGGGGCTTGCAAAGGATCCAG
ACCAGGCTGCAATACTGAAGGTGAAGGAGATCAAGAACGGTAGACTTGCAATGTTTGCAATGCTCG
GTTTCTTCATCCAAGCTTATGTAACGGGAGAAGGTCCAGTTGAAAACCTCGCCAAACATCTAAGCGA
TCCGTTTGCAAACAACTTGCTCACTGTGCTTGCTGGATCTGCTGAAAGAGCTCCTACCCTGTGAATCA
ATGCATGTGCCTTTGATCTGAGTAC

② *CsCP1* corresponding sequence (comp59744_c0_seq1) from the transcriptome induced by mechanical damage (GEFQ00000000, Li *et al.* 2018)**.** But KY709676 does not show differential expression in the transcriptome induced by mechanical damage.

>**comp59744_c0_seq1** (1154 bp)

TACATTATTTTCATCACTTTCATTCATTCACATTTTTAATTTTCAAAAGCAAAGGTAGAGCAAAGCAG
AGCAATGGCTTCACTGGCAGCATCAACGGCGGCTGCCTCCCTTGGCATGTCAGAAATGCTCGGAAAC
CCTCTCCGGAGTGGCGTAACGAGATCGGCACCTCCTCCCACCGCGACATCTAGCCCTGCCACCTTCA
AGACCGTCGCACTTTTCTCCAAGAAGAAGGCTGCACCTCCCAAAAAGGCTGTCGTCTCCCCTGTTGAT
GACGAGCTCGCCAAGTGGTACGGTCCCGACAGAAGAATTTTCTTGCCGGAGGGGCTGTTGGACCGAT
CAGAAATTCCGGCATACCTCACCGGAGAAGTCCCTGGAGATTATGGTTACGATCCTTTTGGGCTTAG
CAAGAAACCAGATGACTTTGCCAAGTACCAAGCATATGAGCTAATTCATGGCAGGTGGGCAATGTTG
GGGGCTGCTGGCTTCATCATCCCTGAGGCCTTCAACAAATTTGGTGCTAACTGTGGCCCTGAAGCTGT
TTGGTTCAAGACAGGAGCTCTACTCCTAGATGGTAACACACTGAATTACTTTGGAAAGAACATCCCC
ATTAATCTTATATTCGCTGTCATCGCTGAAGTTGTTCTTGTTGGTGGTGCTGAATACTACAGAATCAT
CAATGGATTGAATTTGGAGGACAAGCTTCACCCAGGCGGTCCATTCGATCCATTGGGGCTTGCAAAG
GATCCAGACCAGGCTGCAATACTGAAGGTGAAGGAGATCAAGAACGGTAGACTTGCAATGTTTGCA
ATGCTCGGTTTCTTCATCCAAGCTTATGTAACGGGAGAAGGTCCAGTTGAAAACCTCGCCAAACATC
TAAGCGATCCGTTTGCAAACAACTTGCTCACTGTGCTTGCTGGATCTGCTGAAAGAGCTCCTACCCTG
TGAATCAATGCATGTGCCTTTGATCTGAGTACTTTTGAATGAACATTATTGTAACTACCAAGTACTTG
ATTAGACAGACATGTTTCTGAATTCTGATTTAGTTAATCACCTTTGCAATATTGGAGTACTCTTGCAC
TTCTCAATATTTCTCTCAATTTTGAGGCACTTGACCTTCATCTATTCATATATAATCTTAAAAGCTAAA
AAAAAAA

③ *CsCP1* corresponding sequence (CSA004532) from the genome in tea plant (Xia et al. 2017)

>**CSA004532** (972 bp)

ATGGCTTCACTGGCAGCATCAACGGCGGCTGCCTCCCTTGGCATGTCAGAAATGCTCGGAAACCCTC
TCCGGAGTGGCGTAACGAGATCGGCACCTCCTCCCACCGCGACATCTAGCCCTGCCACCTTCAAGAC
CGTCGCACTTTTCTCCAAGAAGAAGGCTGCACCTCCCAAAAAGGCTGTCGTCTCCCCCGTTGATGAC
GAGCTCGCCAAGTGGTACGGTCCCGACAGAAGAATTTTCTTGCCGGAGGGGCTGTTGGACCGATCAG
AAATTCCGGCATACCTCACCGGAGAAGTCCCTGGAGATTATGGTTACGATCCTTTTGGGCTTAGCAA
GAAACCAGATGACTTTGCCAAGTGGGCAATGTTGGGGGCTGCTGGCTTCATCATCCCTGAGGCCTTC
AACAAATTTGGTGCTAACTGTGGCCCTGAAGCTGTTTGGTTCAAGACAGGAGCTCTACTCCTAGATG
GTAACACACTGAATTACTTTGGAAAGAACATCCCCATTAATCTTATATTCGCTGTCATCGCTGAAGTT
GTTCTTGTTGGTGGTGCTGAATACTACAGAATCATCAATGGATTGGTATGCGGTCCATTTGATCCATT
GGGGCTTGCAAAGGATCCAGACCAGGCTGCAATACTGAAGGTGAAGGAGATCAAGAACGGTAGACT
TGCAATGTTTGCAATGCTCGGTTTCTTCATCCAAGCTTATAATTTGGAGGACAAGCTTCACCCAGGCG
GTCCATTTGATCCATTGGGGCTTGCAAAGGATCCAGACCAGGCTGCAATACTGAAGGTGAAGGAGAT
CAAGAACGGTAGACTTGCAATGTTTGCAATGCTCGGTTTCTTCATCCAAGCTTATGTAACGGGAGAA
GGTCCAGTTGAAAACCTCGCCAAACATCTAAGCGATCCGTTTGCAAACAACTTGCTCACTGTGCTTGC
TGGATCTGCTGAAAGAGCTCCTACCCTGTGA

④ Alignment of *CsCP1* with the assembly sequence of tea genome (PRJNA381277, Xia *et al.* 2017) and trauma induced transcriptome (GEFQ00000000,Li *et al*. 2018), Please see the Supplementary Figure S1.

(2) ***CsCP2*** (KY709677, 830 bp)，The identity is 99% (559/560) between *CsCP2* and comp56954_c0_seq1 (977 bp), but *CsCP2* is 270 bp longer at 5' end and 417 bp shorter at 3' end. The sequence consistency is also 99% (793/798) between *CsCP2* and CSA019572. But *CsCP2* is 14 bp at 5 'end 18 bp at 3' end lenger than CSA019572.

① The sequence of ***CsCP2*** was amplified by PCR and submitted to the Genbank

GCTCTAGAGCAGTCATGGCCACCTCTGCTATCCAACAGTCGGCTTTCGCTGGACAGACTGCCCTGAA
GCCCCAGAATGAGCTTGTCAGGAAGATTGGCAGCTTTGGAGGTGGCCGCATCACCATGAGGCGCACT
GTGAGAAGTGCTCCCCAAAGCATATGGTATGGGCCAGACCGCCCAAAGTACTTGGGCCCATTCTCCG
AGCAAACTCCATCATACTTGACCGGTGAATTCCCCGGTGACTATGGTTGGGACACTGCTGGGCTTTCA
GCAGACCCCGAGACCTTTGCCAAGAACCGTGAACTCGAGGTGATCCACTGCCGCTGGGCCATGCTCG
GTGCACTGGGCTGTGTCTTCCCCGAAATCCTTTCCAAGAATGGTGTCAAGTTCGGTGAGGCAGTTTGG
TTCAAGGCTGGAGCCCAAATTTTCTCCGAAGGTGGCCTTGATTACCTTGGCAATCCAAACCTGATCCA
TGCCCAGAGCATCCTCGCAATCTGGGCTTGCCAAGTAGTGCTCATGGGCTTCATTGAGGGCTACCGG
GTTGGCGGAGGCCCACTTGGCGAAGGACTCGTCAAAATCTACCCCGGAGGAGCCTTTGACCCACTTG
GACTTGCTGATGATCCAGAAGCATTTGCAGAGTTGAAGGTGAAGGAGATCAAGAATGGACGGCTGG
CGATGTTTTCAATGTTTGGATTCTTCGTCCAGGCCATCGTTACTGGAAAGGGCCCAATTGAGAACCTC
TTCGACCATCTCGCTGATCCTGTGGCCAACAATGCTTGGGCTTATGCCACTAACTTCGTACCTGGAAA
ATGAAGTATGAAGATTGAGCTG

② ***CsCP2*** corresponding sequence **(**comp56954_c0_seq1**)** from the transcriptome induced by mechanical damage (GEFQ00000000, Li *et al*. 2018)

>**comp56954_c0_seq1** (977 bp)
CAGACCCCGAGACCTTTGCCAAGAACCGTGAACTCGAGGTGATCCACTGCCGCTGGGCCATGCTCGG
TGCACTGGGCTGTGTCTTCCCCGAAATCCTTTCCAAGAATGGTGTCAAGTTCGGTGAGGCAGTTTGGT
TCAAGGCTGGAGCCCAAATTTTCTCCGAAGGTGGCCTTGATTACCTTGGCAATCCAAACCTGATCCAT
GCCCAGAGCATCCTCGCAATCTGGGCTTGCCAAGTAGTGCTCATGGGCTTCATTGAGGGCTACCGGG
TTGGCGGAGGCCCACTTGGCGAAGGACTCGACAAAATCTACCCCGGAGGAGCCTTTGACCCACTTGG
ACTTGCTGATGATCCAGAAGCATTTGCAGAGTTGAAGGTGAAGGAGATCAAGAATGGACGGCTGGC
GATGTTTTCAATGTTTGGATTCTTCGTCCAGGCCATCGTTACTGGAAAGGGCCCAATTGAGAACCTCT
TCGACCATCTCGCTGATCCTGTGGCCAACAATGCTTGGGCTTATGCCACTAACTTCGTACCTGGAAAA
TGAAGTATGAAGATTGAGCTGTAACATTACTTAAAGCTTCTTGGGGTAATTGAATGTGTATTGGGTCC
ATCCTGATGTAATATTACTTGCAGAAACTGCCTATCTGAGACTTTTATTGCCACTTTTGAGTGTTCAG
ATCTTATTGTTGCTATAATTTTCATCTTCTCTTCAACATTAAGCTGGCACAGGTATCTGCACAAAGTA
AACCCAACTTAAACATAGCTTTATGTTCACTTTGGCAGCTAAGAGTGAACAACAAAAGCAAAATTGA
ACTATGAAAGTGCAGTTCCAAACCCATACAGTGTGATGGATTTCATCAAGTAAACCAATTGCTATTG
GCAACTATTTGTTAATATCAAATTTGCAACTTAAAATGCTTGTCCGCATTCCCATAATGAATGCATAC
ATCCATTAGGCCAATTAACTGAAATCTTCTGG

The FPKM of comp56954_c0_seq1 in the transcriptome induced by mechanical damage：

Sampling point in time	0 h	3 h	6 h	12 h	24 h
comp56954_c0_seq1 RPKM：	3660.4 （1.0）	2741.9 （0.75）	2698.43 （0.74）	147.94 （0.04）	2644.75 （0.72）

③ ***CsCP2*** corresponding sequence (CSA019572) from the genome in tea plant (Xia et al. 2017)

>**CSA019572** (798 bp)

ATGGCCACCTCTGCTATCCAACAGTCGGCTTTCGCTGGACAGACTGCCCTGAAGCCCCAGAATGAGC
TTGTCAGGAAGATCGGCAGCTTCGGAGGTGGCCGCATCACCATGAGGCGCACTGTGAGAAGTGCTCC

CCAAAGCATATGGTATGGGCCAGACCGCCCAAAGTACTTGGGCCCATTCTCCGAGCAAACTCCATCA
TACTTGACCGGTGAATTCCCCGGTGACTATGGTTGGGACACTGCTGGGCTTTCAGCAGACCCCGAGA
CCTTTGCCAAGAACCGTGAACTCGAGGTGATCCACTGCCGCTGGGCCATGCTCGGTGCACTGGGCTG
TGTCTTCCCCGAAATCCTCTCCAAGAATGGTGTCAAGTTCGGTGAGGCAGTTTGGTTCAAGGCTGGA
GCCCAAATTTTCTCCGAAGGTGGCCTTGATTACCTTGGCAATCCAAACCTGATCCATGCCCAGAGCAT
CCTTGCAATCTGGGCTTGCCAAGTAGTGCTCATGGGCTTCATTGAGGGCTACCGGGTTGGCGGAGGC
CCACTTGGCGAAGGACTCGACAAAATCTACCCCGGAGGAGCCTTTGACCCACTTGGACTTGCTGATG
ATCCAGAAGCATTTGCAGAGTTGAAGGTGAAGGAGATCAAGAATGGACGGCTGGCGATGTTTTCAAT
GTTTGGATTCTTCGTCCAGGCCATCGTTACTGGAAAGGGCCCAATTGAGAACCTCTTCGACCATCTCG
CTGATCCTGTGGCCAACAATGCTTGGGCTTATGCCACTAACTTCGTACCTGGAAAATGA

④ Alignment of CsCP2 with the assembly sequence of tea genome (PRJNA381277, Xia *et al.* 2017) and trauma induced transcriptome (GEFQ00000000,Li *et al*. 2018), Please see the Supplementary Figure S1.

2) Table S2. Domain analysis of 25 CAB proteins in tea plant

>CSA030474.1

MAASTMALSSPSFAGKAIKLSPSTPDLVGRGRISMRKTGGKPVRSGSPWYGPDRVLYLGPLSGDPPSYLTG
EFPGDYGWDTAGLSADPETFSKNRELEVIHCRWAMLGALGCVFPELLAGNGVKFGEAIWFKAGAQIFSE
GGLDYLGNPSLIHAQSILAIWACQVILMGAVEGYRIAGGPLGEVTDPLYPGGSFDPLGLADDPEAFAELKV
KEIKNGRLAMFSMFGFFVQAIVTGKGPLENLADHLADPVNNNAWAYATNFVPGNIVLSNGLFGYRL*

（There is a chloroplast transporter peptide at the N-terminal of the protein. The underlined part from 65-232 aa refers to chlorophyll a/b binding domain which contains three transmembrane α-helical regions being marked with frames. Additionally, the 67-135 aa segment in the protein contains a RHO domain）

>CSA030476.1

MAASTMALSSPSFAGKAVKLSPSTPDLVGRGRISMRKSFGKPGPSGSPWYGPDRVLYLGPLSGEPPSYLTG
EFPGDYGWDTAGLSADPETFAKNRELEVIHCRWAMLGALGCVFPELLARNGVKFGEAIWFKAGAQIFSE
GGLDYLGNPGLIHAQSILAIWACQVILMGAVEGYRIAGGPLGEVTDPLYPGGSFDPLGLADDPEAFAELKV
KEIKNGRLAMFSMFGFFVQAIVTGKGPLENLADHLADPVNNNAWAYATNFVPGK*

（There is a chloroplast transporter peptide at the N-terminal of the protein. The underlined part from 65-232 aa refers to chlorophyll a/b binding domain which contains three transmembrane α-helical regions being marked with frames. Additionally, the 67-135 aa segment in the protein contains a RHO domain）

>CSA008917.1, 261 aa

MAASTMALSSPSFAGKAVKLAPEVLGGGRISMRKTGKQVPSGSPWYGPDRVLYLGPLSGDPPSYLTGEFP
GDYGWDTAGLSADPETFAKNRELEVIHCRWAMLGALGCVFPELLARNGVKFGEAVWFKAGAQIFSEGGL
DYLGNPSLIHAQSILAIWACQVILMGAVEGYRIAGGPLGEVTDPLYPGGSFDPLGLADDPEAFAELKVKEI
KNGRLAMFSMFGFYVQAIVTGKGPLENLADHLADPVNNNAWAYATNFVPGK*

[There is a chloroplast transporter peptide at the N-terminal of the protein. The underlined part from 61-228 aa refers to chlorophyll a/b binding domain which contains three transmembrane α-helical regions being marked with frames. Additionally, the212-259 aa segment (blue) in the protein contains a phosphatase domain]

>CSA035910.1

MRKTGKQVPSGSPWYGPDRVLYLGPLSGEPPSYLTGQFPGDYGWDTAGLSADPETFAKNRELEVIHCRW
AMLGALGCVFPELLARNGVKFGEAVWFKAGAQIFSEGGLDYLGNPSLIHAQSILAIWACQVILMGAVEGY

RIAGGPLGEVTDPLYPGGSFDPLGLADDPEAFAELKVKEIKNGRLAMFSMFGFFDQAIVTGKGPLENLAD HLADPVNNNAWAYATNFVPGK*

（There is no a chloroplast transporter peptide at the N-terminal of the protein. The underlined part from 30-197 aa refers to chlorophyll a/b binding domain which contains three transmembrane α-helical regions being marked with frames）

>CSA019572.1, 265 aa

MATSAIQQSAFAGQTALKPQNELVRKIGSFGGGRITMRRTVRSAPQSIWYGPDRPKYLGPFSEQTPSYLTGE FPGDYGWDTAGLSADPETFAKNRELEVIHCRWAMLGALGCVFPEILSKNGVKFGEAVWFKAGAQIFSEG GLDYLGNPNLIHAQSILAIWACQVVLMGFIEGYRVGGGPLGEGLDKIYPGGAFDPLGLADDPEAFAELKV KEIKNGRLAMFSMFGFFVQAIVTGKGPIENLFDHLADPVANNAWAYATNFVPGK*

[There is a chloroplast transporter peptide at the N-terminal of the protein. The underlined part from 65-232 aa refers to chlorophyll a/b binding domain which contains three transmembrane α-helical regions being marked with frames. Additionally, the219-261 aa segment (blue) in the protein contains a phosphatase domain]

>CSA035674.1

MLGALGCITPEVLEKWVKVDFKEPVWFKAGAQIFSKGGLDYLGNPNLVHAQSILAVLGFQVILMGLVEG FRINGLPGVEKGNNLYPGGQYFDPLGLADDPVTFAELKVKEIKNGRLAMFSMFGFFVQAIITNKGPLENL LDHLDNPVANNAWVYHLCHQVCTRVINS*

（There is no a chloroplast transporter peptide at the N-terminal of the protein. The underlined part from 1-129 aa refers to chlorophyll a/b binding domain which contains two transmembrane α-helical regions being marked with frames）

>CSA019509.1

MLRALGCITPEVLEKWVKMDFKKPGSFKSGAQIFSESGLHYLGNPNLVHTQSILAVLGFQVVLMGLVEGF HINGLPRVEEGNNLYPGGQYFDPLGLADDAVTFAELKVKEIRNGRLAMFSTFGFFVQAIVTSKGPLENLLD HLDNPVANNAWVYATKPIFDRRAGVLIDDLPQSYPSSRAEATLVMTDTWP*

（There is no a chloroplast transporter peptide at the N-terminal of the protein. The underlined part from 1-129 aa refers to chlorophyll a/b binding domain which contains two transmembrane α-helical regions being marked with frames）

>CSA002361.1

MASMLMAATASTSTALRPTPFLGQTRGPNANPLRDVVSMGTGKFTMGNELWYGPDRLKYLGPFSAQTPS YLTGEFPGDYGWDTAGLSADPEAFARNRALEKIEKPMGEKNVKVIHGRWAMLGALGCITPEVLQKWVRV AFKEPVWFKAGAQIFSEGGLDYLGNPNLVHAQSILAVLGFQVVLMGLVEGFRINGLPGVGEGNNLYPGGQ YFDPLGLADDPVTFAELKVKEIKNGRLAMFSMFGFFVQAIVTGKGPLENLLDHLENPVANNAWVYATKF VPGS*

（There is a chloroplast transporter peptide at the N-terminal of the protein. The underlined part from 67-248 aa refers to chlorophyll a/b binding domain which contains three transmembrane α-helical regions being marked with frames. Additionally, the 69-122 aa segment in the protein contains a RHO domain）

>CSA016997.1

MATMSTATSTSTVLRPTPFLGQTRGANANPLRDVVSMGIGKFTMGNELWYGPDRVKYLGPFSAQTPSYLT GEFPGDYGWDTAGLSADPEAFARNRALEVIHGRWAMLGALGCITPEVLEKWVKVDFKEPVWFKAGAQI

FSEGGLDYLGNPNLVHAQSILAVLGFQVVLMGLVEGFRINGLPGVGEGNNLYPGGQYFDPLGLADDPVTFAELKVKEIKNGRLAMFSMFGFFVQAIVTGKGPLENLLDHLDNPVANNAWVYATKFVPGS*

（There is a chloroplast transporter peptide at the N-terminal of the protein. The underlined part from 65-234 aa refers to chlorophyll a/b binding domain which contains three transmembrane α-helical regions being marked with frames）

>CSA004532.1

MASLAASTAAASLGMSEMLGNPLRSGVTRSAPPPTATSSPATFKTVALFSKKKAAPPKKAVVSPVDDELAKWYGPDRRIFLPEGLLDRSEIPAYLTGEVPGDYGYDPFGLSKKPDDFAKWAMLGAAGFIIPEAFNKFGANCGPEAVWFKTGALLLDGNTLNYFGKNIPINLIFAVIAEVVLVGGAEYYRIINGLVCGPFDPLGLAKDPDQAAILKVKEIKNGRLAMFAMLGFFIQAYNLEDKLHPGGPFDPLGLAKDPDQAAILKVKEIKNGRLAMFAMLGFFIQAYVTGEGPVENLAKHLSDPFANNLLTVLAGSAERAPTL*

（There is a chloroplast transporter peptide at the N-terminal of the protein; The chlorophyll a_b binding domains consisted of two fragment from 91-237 aa and 238-287 aa, which was marked with underline; three frame regions refer to the transmembrane α-helical region.）

The align results of the assembly sequence with sequencing sequence:

```
CsCP1  1    MASLAASTAAASLGVSEMLGNPLRSGVTRSAPPPTATSSPATFKTVALFSKKKAAPPKKAVVSPVDDELAKWYGPDRRIFLPEGLLDRSEIPAYLTGEVPGDYGYDPFGLSKKPDDFAKY  120

Sbjct  1    MASLAASTAAASLGMSEMLGNPLRSGVTRSAPPPTATSSPATFKTVALFSKKKAAPPKKAVVSPVDDELAKWYGPDRRIFLPEGLLDRSEIPAYLTGEVPGDYGYDPFGLSKKPDDFAK-  119


CsCP1  121  QAYELIHGRWAMLGAAGFIIPEAFNKFGANCGPEAVWFKTGALLLDGNTLNYFGKNIPINLIFAVIAEVVLVGGAEYYRIINGL------------------------------------  204

Sbjct  120  ---------WAMLGAAGFIIPEAFNKFGANCGPEAVWFKTGALLLDGNTLNYFGKNIPINLIFAVIAEVVLVGGAEYYRIINGLVCGPFDPLGLAKDPDQAAILKVKEIKNGRLAMFAML  230


CsCP1  205  -------NLEDKLHPGGPFDPLGLAKDPDQAAILKVKEIKNGRLAMFAMLGFFIQAYVTGEGPVENLAKHLSDPFANNLLTVLAGSAERAPTL  290

Sbjct  231  GFFIQAYNLEDKLHPGGPFDPLGLAKDPDQAAILKVKEIKNGRLAMFAMLGFFIQAYVTGEGPVENLAKHLSDPFANNLLTVLAGSAERAPTL  323
```

Figure The align result of the proteins encoded *CsCP1* and CSA004532.1 from the genome data of tea plants

>CSA003567.1

MAMLQQSSISSSGLLSKGAAPKSLNIYTNSQPLAKPYRACKASWQELVGVLIFSAVPFTAVKAIANSPLGELLQRRLEEKKKDAIDNSSNFKALAQMARKDSLWYGEKRPRWLGPISYDYPSYLTGELPGDYGFDIAGLSRDPVAFQKYFNFEILHARWAMLAALGALIPELLDLVGAFHFVEPVWWKVGYSKLKGDTLDYLGIPGLHLAGSQGVVVIAICQALLMGFGVTIEGPD*

（There is a chloroplast transporter peptide at the N-terminal of the protein. The underlined part from 120-233 aa refers to chlorophyll a/b binding domain which contains two transmembrane α-helical regions being marked with frames）

>CSA016587.1

MATQALVSSSSLTSSVEAARQILGGRPATHSSRRKVSFVVRAATTPPVKQGADRPLWFASKQSLTYLDGSLPGDYGFDPLGLSDPEGTGGFIEPRWLAYGEIFNGRTAMVGSIGCIAPEILGKLGLIPPETALPWFKTGVIPPAGTYDYWADPYTLFVFELALVGFAEHRRFQAWYNPGSMGKQYFLGLEKYLGGTDNPAYPGGPLFNPLGLGKDEKSMRDMKLKEVKNGRLAMLGMLGFFVQALVTGVGPFQNLLDHLADPVNNNVLTNLKFH*

（There is a chloroplast transporter peptide at the N-terminal of the protein. The underlined part from 65-234 aa refers to chlorophyll a/b binding domain which contains three transmembrane α-helical regions being marked with frames）

>CSA024064.1

MASKALMSCGIAAVCPSVLSSSKSKFAAALPLPSGGATATSRLTMTADWMPGEPRPPYLDGSAPGDFGFDPLRLGEVPENLERYKESELIHCRWAMLAVPGILVPEALGLGNWVQAQEWAAIPGGQATYLGQPVPWGTLPIILAIEFLAISFVEHQRSMEKDPEKKKYPGGAFDPLGYSKDPVKFEENKVKEVKNGRLALLAFVGICVQQSAYPGTGPLENLATHLADPWHNNIGDIIIPRSISP*

（There is a chloroplast transporter peptide at the N-terminal of the protein. The underlined part from 55-211 aa refers to chlorophyll a/b binding domain which contains three transmembrane α-helical regions being marked with frames. Additionally, the 69-122 aa segment in the protein contains a RHO domain）

>CSA035688.1

MAATSAPVGSPFLSGGKRSQTLLLSATIGARSLGGGVVAPKKFVLLAAALKKSWIPAVKGGGSFINPEWLDDSLPGDYGFDPLGLGKDPAFLKWYREAELIHGRWAMAAVVGIFVGQAWSGIPWFEAGADPSAIAPFSFGTLLGTQLLLMGWVESKRWVDFFNPESQSVEWATPWSRTAENFANATGDQGYPGGKFFDPLCLAGTIVNGVYVPDTEKLERLKLAEIKHARIAMSAMLIFYFEAGQGKTPLGALGL*

（There is a chloroplast transporter peptide at the N-terminal of the protein. The underlined part from 60-244 aa refers to chlorophyll a/b binding domain which contains three transmembrane α-helical regions being marked with frames）

>CSA008855.1

MNFQFNHREWWSSGEALALYADRHLAMIGAGESGPPGLGLRRLPGDYGFDPLGLGKDPAFLKWYREAELIHGRWAMAAVVGIFVGQAWSGIPWFEAGADPGAIAPFSFGTLLGTQLLLMGWVESKRWVDFFNPESQSVEWATPWSKTAENFANATGDQGYPGGKFFDPLCLAGTIVNGVYIPDKEKLDRLKLAEIKHARLAMVAMLIFYFEAGQGKTPLGALGL*

（There is no a chloroplast transporter peptide at the N-terminal of the protein. The underlined part from 36-213 aa refers to chlorophyll a/b binding domain which contains three transmembrane α-helical regions being marked with frames）

>CSA014653.1

MAATTSAAATSSFIGTRFPEVHSGSGRVQARFGFGAKKAPAKKFAKPGSDRPLWFPGAKAPEWLDGTLVGDYGFDPFGLGKPAEYLQFELDSLDQNLAKNLAGDIIGTRFESADVKSTPFQPYSEVFGLQRFRECELIHGRWAMLATLGALTVEWLTGITWQDAGKVELLEGSSYLGQPLPFSITTLIWIEVLVIGYIEFQRNSELDPEKRLYPGGKYFDPLGLASDPEKKATLQLAEIKHARLAMIAFLGFAVQAAATGKGPLNNWATHLSDPLHTTIIDTFFS*

（There is no a chloroplast transporter peptide at the N-terminal of the protein. The underlined part from 60-257 aa refers to chlorophyll a/b binding domain which contains three transmembrane α-helical regions being marked with frames）

>CSA010862.1

MAATTAAAAAATSSFLGTRLADLCSGSGRVQARFGFGRKKAPPKKIAKQGFDRPLWFPGAKAPEWLDGSLVGDYGFDPFGLGKPAEYLQFDLDSLDQNLAKNSAGDVIGTRFESADVKSTPFQPYTEVFGLQRFRECELIHGRWAMLATLGALTVEWLTGVTWQDAGKVELIEGSSYLGQPLPFSITTLIWIEVIVIGYIEFQRNAELDPEKRLYPGGKFFDPLGLASDPEKKATLQLAEIKHARLAMVAFLGFAVQAAVTGKGPLNNWATHLSDPLHTTIIDTFFS*

（There is no a chloroplast transporter peptide at the N-terminal of the protein. The underlined part from 62-259 aa refers to chlorophyll a/b binding domain which contains three transmembrane α-helical regions being marked with frames）

>CSA032834.1

MATITAQASTAVFRPRAAKSQFLTGSSGKLNREISLKSKSSSPRSFKVEAKGEWLPGLPSPDYLNGSLPGDNGFDPLGLAEDPENLKWYIQAELVNSRWAMLGVTGMLLPEVFSTIGIINVPKWYDAGKAEYFASSSTLFVIEFILFHYVEIRRWQDIKNPGSVNQDPIFKNYSLPPGEVGYPGGIFNPLNFAPTQEAKEKELANGRLAMLAFLGFIVQHNVTGKGPFDNLLQHLSDPWHNTIIQTFQGY*

（There is a chloroplast transporter peptide at the N-terminal of the protein. The underlined part from 60-221 aa refers to chlorophyll a/b binding domain which contains three transmembrane α-helical regions being marked with frames）

>CSA012994.1

LPGDNGFDPLGLAEDPENLKWFIQAELVNSRWAMLGVTGMLLPEVLSSIGIINVPKWYDAGKSEYFASSSTLFVIEFILFHYVEIRRWQDIKNPGSVNQDPIFKSYSLPPNECGYPGGIFNPLNFAPTEEAKEKELANGKFLKYHVTLL*

（There is no a chloroplast transporter peptide at the N-terminal of the protein. The underlined part from 1-141 aa refers to chlorophyll a/b binding domain which contains two transmembrane α-helical regions being marked with frames）

>CSA020482.1

MALSIASTALSSLPIRKIPGKAPGKIATGLAWKTSVNATKGGVSSVCEPLPPDRPLWFPGSSPPEWLDGSLPGDFGFDPLGLGSDPELLKWFAQAELMHARWAMLAVAGILIPEWLESLELIENFSWYDAGAREYFADPTTLFVVQLVLMGWVEGRRWADMVNPGCVDIEPTYPTKKKPKADVGYPGGLWFDPFMWGRGSPEPVMVLRTKEIKNGRLAMLAFVGFCFQAIYTGQGPIENLMAHIADPGHCNIFSVRRL*

（There is a chloroplast transporter peptide at the N-terminal of the protein. The underlined part from 63-230 aa refers to chlorophyll a/b binding domain which contains three transmembrane α-helical regions being marked with frames）

>CSA011328.1

MYSPIHQDKPLENPYIKQPLISLIHTNQSNTQNSSKENQPNMASSAFASSAIAAVGLSSPSSKKSGSIVGATKASFFGGRKLRLRKYSTSPAGARSVTVCVAADPDRPIWFPGSVPPPWLDGSLPGDFGFDPLGLGSDPETLRWNVQSELVHCRWAMLGAAGIFIPEFLTKIGILNTPSWYTAGELEYFTDTTTLFIIELILIGWAEGRRWADILKPGCVNTDPIFPNNKLTGTDVGYPGGLWFDPLGWGSGSPEKVKELRTKEIKNGRLAMLAVMGAWFQAIYTGTGPIDNLFAHLADPGHATIFAVSAF*

（There is no a chloroplast transporter peptide at the N-terminal of the protein. The underlined part from 116-283 aa refers to chlorophyll a/b binding domain which contains three transmembrane α-helical regions being marked with frames）

>CSA011380.1

MASSVCASSAFAAVGLSSPSSQKSGSIVGATKASFPGGRKLRLRKNAAPAGTPSGSVTVCVAADPHRPLWFPGNTPPPWXLVKWDKHTDNAQCLSHKSGSIVGAAKASFLGGRKLRLRKNAAPAGTPSGSVTVCVAVDPDRPLWFPGSTPPPWLDGSCYVTEVVAEPCDAASLETLDLIHLVLISKSFEPLLTGSDPETLRWNVQAELVHCR

WAMLGAAGIFIPEFLTKLGILNTPSWYTAGEQEYFTDTTTLFIVELIFIGWAEGRRWADILKPGCVNTDPIFP
NNKLTGTDVGYPGGLWFDPLGWGSSSPEKIRELRTKEIKNGRLAMLDVMGAWFQAIYTGTGPIDNLFAHL
ADPGLHPQVIGASEQAC*

（There is a chloroplast transporter peptide at the N-terminal of the protein. The underlined part from 172-341 aa refers to chlorophyll a/b binding domain which contains three transmembrane α-helical regions being marked with frames）

>CSA013374.1

MAQTMLLTSSSSVNTHALDLKRQPLLESLRPKPFSHILLPPLPSSSSFHQTTTIALFKSKTKAAPVKKVAEPK
PKVESGIFGTSGGIGFTKQNELFVGRVAMIGFAASLLGEAITGKGILAQLNLETGIPIYEAEPLLLFFILFTLL
GAIGALGDRGRFVDDPPTGLDKAVIPPGKSLRSALGLKEGGPLFGFTKSNELFVGRLAQLGIAFSIIGEIITG
KGALAQLNIETGVPISDIEPLVLFNVLFFFVAALNPGTGKFQTDEEE*

（There is a chloroplast transporter peptide at the N-terminal of the protein. The underlined part from 57-262 aa refers to chlorophyll a/b binding domain which contains two transmembrane α-helical regions being marked with frames）

>CSA016009.1

MATSAIMQSVLARPVTSVTTRARFGQFTPCSYVPYLQKNASMQVRCMAKYGQKEDVTPKPKVSTKFSDV
LAFSGPAPERINGRLAMIGFVAAMAVELSNGEDVLVQISNGGVPWFVGTSIVLTLASLVPLFKGVSVESRSE
RIMSSDAELWNGRFAMLGLVALAFTEFVKGGALV*

（There is a chloroplast transporter peptide at the N-terminal of the protein. The underlined part from 64-147 aa refers to chlorophyll a/b binding domain which contains two transmembrane α-helical regions being marked with frames）

>CSA016010.1

MATSAMQSILARPVTGVTTRARFSQSTPCSYVPRLRRNAGMQVRCMAEDGQNKDPTPTTTPPPASQPIPSP
PPKPKVSTKFSDVLAFSGPAPERINGRLAMIGFVAAMAVELSKGEDVLAQISNGGVSWFVGTSIVLTLASLI
PLFKGVSVESRSEGIMSSDAELWNGRFAMLGLVALAFTEFVKGGALV*

（There is a chloroplast transporter peptide at the N-terminal of the protein. The underlined part from 74-183 aa refers to chlorophyll a/b binding domain which contains two transmembrane α-helical regions being marked with frames）

3) Table S3. CAB gene identifiers in the tea plant genome

length(aa)	Gene identifiers		Chromosomal locations	
73	CSA003566.1	CSA003566	Sc0000791:117456-119034(-)	Too short
149	CSA012994.1	CSA012994	xfSc0045437:285-734(+)	
167	CSA035674.1	CSA035674	xfSc0001566:7779-8282(-)	
175	CSA016009.1	CSA016009	Sc0001400:437624-439288(-)	
190	CSA016010.1	CSA016010	Sc0001400:467079-469111(-)	
191	CSA019509.1	CSA019509	Sc0003165:80030-84657(-)	
224	CSA008855.1	CSA008855	Sc0001913:24353-25428(-)	
230	CSA035910.1	CSA035910	xpSc0054180:92847-93539(+)	
236	CSA003567.1	CSA003567	Sc0000791:120524-124674(-)	

245	CSA024064.1	CSA024064	Sc0003245:116483-118990(+)	
250	CSA032834.1	CSA032834	Sc0000132:871603-873334(-)	
255	CSA035688.1	CSA035688	Sc0000727:56215-57155(-)	
258	CSA020482.1	CSA020482	Sc0005399:78226-80137(-)	
261	CSA008917.1	CSA008917	Sc0000033:1432177-1432962(+)	
265	CSA030476.1	CSA030476	xpSc0053783:87485-88282(-)	
**265**	**CSA019572.1**	**CSA019572**	**Sc0000527:191398-193018(+)**	***CsCP2***
267	CSA016997.1	CSA016997	xpSc0055362:18915-20179(+)	
267	CSA013374.1	CSA013374	Sc0000308:701499-704468(+)	
274	CSA016587.1	CSA016587	Sc0001083:148540-150625(+)	
277	CSA030474.1	CSA030474	xpSc0053783:90029-91192(+)	
281	CSA002361.1	CSA002361	Sc0001464:12875-14696(-)	
285	CSA014653.1	CSA014653	Sc0003119:194472-195756(+)	
287	CSA010862.1	CSA010862	xfSc0000803:95575-98020(-)	
311	CSA011328.1	CSA011328	Sc0000700:318486-321172(+)	
**323**	**CSA004532.1**	**CSA004532**	**Sc0003157:144877-147574(+)**	***CsCP1***
372	CSA011380.1	CSA011380	Sc0002776:277155-279299(-)	
898	CSA030398.1	CSA030398	Sc0004691:24118-29708(-)	Too long

4) Table S4. CDSs on 25 CAB genes in the tea plant genome

>CSA030474.1
ATGGCTGCCTCTACAATGGCTCTCTCTTCTCCATCTTTCGCCGGAAAGGCGATAAAACTCTCTCCTTCCACCCCAGACCTCGT
TGGCAGAGGAAGGATCAGCATGAGGAAGACTGGTGGCAAGCCCGTCCGATCCGGTAGCCCATGGTACGGCCCAGACCGAG
TCTTGTATTTGGGCCCACTCTCTGGTGACCCCCCATCCTACCTGACTGGAGAATTCCCTGGTGACTACGGTTGGGACACTGCT
GGGCTTTCAGCTGACCCAGAAACATTTTCCAAGAACCGTGAGCTCGAGGTGATCCATTGCAGATGGGCCATGCTCGGCGCT
CTTGGGTGTGTCTTCCCCGAGCTTTTGGCCGGCAATGGTGTCAAGTTCGGTGAGGCTATATGGTTCAAAGCCGGGGCCCAAA
TCTTCAGTGAGGGTGGGCTTGACTACTTGGGCAACCCTAGCTTGATCCATGCTCAAAGCATTTTGGCCATTTGGGCTTGCCA
AGTTATCTTGATGGGCGCCGTGGAGGGCTACCGTATTGCAGGTGGGCCGCTCGGTGAGGTGACCGACCCACTCTACCCGGGT
GGAAGCTTCGACCCATTGGGCCTTGCCGATGACCCAGAGGCCTTTGCTGAGCTCAAGGTGAAGGAGATCAAGAATGGTAGA
CTTGCCATGTTTTCCATGTTTGGATTCTTTGTTCAAGCCATTGTGACTGGAAAGGGACCATTGGAGAACCTTGCTGACCACCT
TGCTGATCCAGTGAACAACAATGCCTGGGCTTATGCCACTAACTTTGTTCCCGGAAATATTGTACTGTCTAATGGGTTGTTTG
GATACAGGTTGTAA

>CSA030476.1
ATGGCTGCTTCCACAATGGCTCTCTCTTCTCCATCTTTCGCCGGAAAGGCGGTGAAACTCTCTCCTTCCACCCCAGACCTCGT
TGGCAGAGGAAGGATCAGCATGAGGAAGTCTTTTGGCAAGCCAGGCCCATCAGGTAGCCCGTGGTACGGCCCAGACCGGG
TCTTGTACTTGGGCCCACTCTCCGGTGAGCCCCCATCCTACCTCACTGGAGAATTCCCTGGTGATTATGGCTGGGACACTGCT
GGGCTTTCAGCTGATCCAGAAACATTCGCCAAGAACCGTGAGCTTGAGGTGATCCACTGCAGATGGGCCATGCTTGGAGCT
CTTGGGTGTGTCTTCCCAGAGCTCTTGGCCCGCAACGGTGTCAAGTTCGGTGAGGCCATCTGGTTCAAGGCTGGAGCCCAA
ATCTTCAGTGAGGGTGGACTTGACTACTTGGGCAACCCTGGCTTGATCCATGCTCAAAGCATTTTGGCCATTTGGGCTTGCC
AAGTTATCTTGATGGGCGCCGTGGAGGGATACCGTATTGCTGGTGGGCCTCTCGGTGAGGTGACTGACCCACTCTACCCGGG
TGGAAGCTTCGACCCGTTGGGCCTTGCCGATGACCCAGAGGCCTTTGCTGAGCTAAAGGTGAAGGAGATCAAGAATGGTAG
ACTAGCCATGTTTTCCATGTTTGGATTCTTTGTTCAAGCCATTGTGACTGGAAAGGGACCATTGGAGAACCTTGCTGACCACC
TTGCTGATCCAGTGAACAACAATGCTTGGGCTTATGCCACCAACTTTGTCCCCGGAAAATGA

>CSA008917.1
ATGGCTGCTTCTACCATGGCTCTCTCTTCCCCATCTTTCGCCGGAAAGGCGGTGAAACTTGCCCCGGAGGTTCTTGGTGGTG
GAAGGATCAGTATGAGGAAGACCGGCAAGCAAGTCCCATCTGGAAGCCCGTGGTACGGTCCAGACCGAGTCTTGTATTTGG
GTCCATTGTCTGGTGATCCCCCATCTTACCTCACTGGAGAATTCCCTGGTGATTATGGTTGGGACACTGCTGGGCTTTCAGCC
GATCCAGAAACTTTTGCCAAGAACCGTGAGCTTGAGGTGATTCACTGCAGATGGGCCATGCTTGGAGCTTTGGGTTGCGTCT
TCCCCGAGCTTTTGGCCCGCAACGGTGTCAAGTTCGGCGAGGCTGTGTGGTTCAAGGCCGGTGCCCAAATCTTCAGTGAGG
GTGGGCTTGACTACTTGGGCAACCCTAGCTTGATCCATGCCCAAAGCATTCTGGCCATCTGGGCTTGCCAAGTTATCTTGATG
GGGGCTGTTGAGGGTTACCGTATTGCCGGTGGGCCACTAGGAGAGGTGACAGACCCGCTCTACCCCGGCGGAAGCTTTGAC

CCATTGGGCCTAGCTGATGATCCAGAGGCTTTTGCAGAGCTCAAGGTGAAGGAGATCAAGAATGGAAGACTAGCTATGTTCT
CAATGTTTGGGTTCTATGTTCAGGCCATTGTGACAGGAAAGGGACCATTGGAGAACCTGGCTGACCATCTTGCTGACCCAGT
TAACAACAATGCTTGGGCTTATGCTACCAACTTTGTCCCCGGCAAGTGA

>CSA035910.1
ATGAGGAAGACCGGCAAGCAAGTCCCATCTGGAAGCCCATGGTACGGTCCAGACCGAGTCTTGTATTTGGGTCCATTATCTG
GTGAGCCCCCATCCTACCTCACTGGGCAATTCCCTGGTGATTATGGTTGGGACACTGCTGGGCTTTCAGCTGATCCAGAAAC
TTTTGCCAAGAACCGTGAGCTCGAGGTGATTCACTGCAGATGGGCCATGCTTGGAGCTTTGGGTTGCGTCTTCCCAGAGCTT
TTGGCCCGCAACGGTGTCAAGTTCGGTGAGGCTGTGTGGTTCAAGGCCGGTGCCCAAATCTTCAGTGAGGGTGGGCTTGAC
TACTTGGGCAACCCTAGCTTGATCCATGCCCAAAGCATTCTGGCCATCTGGGCTTGCCAAGTTATCTTGATGGGTGCTGTTGA
GGGCTACCGCATTGCTGGTGGACCGCTCGGGGAGGTCACCGACCCGCTCTACCCCGGTGGAAGCTTCGATCCATTGGGCCT
AGCTGATGATCCAGAGGCTTTTGCAGAGCTCAAGGTGAAGGAGATCAAGAACGGAAGACTGGCTATGTTCTCAATGTTTGG
GTTCTTTGATCAGGCAATTGTGACAGGAAAGGGACCATTGGAGAACCTGGCTGACCACCTTGCTGACCCTGTTAACAACAA
TGCATGGGCTTATGCCACAAACTTTGTCCCAGGAAAGTGA

>CSA019572.1
ATGGCCACCTCTGCTATCCAACAGTCGGCTTTCGCTGGACAGACTGCCCTGAAGCCCCAGAATGAGCTTGTCAGGAAGATC
GGCAGCTTCGGAGGTGGCCGCATCACCATGAGGCGCACTGTGAGAAGTGCTCCCCAAAGCATATGGTATGGGCCAGACCGC
CCAAAGTACTTGGGCCCATTCTCCGAGCAAACTCCATCATACTTGACCGGTGAATTCCCCGGTGACTATGGTTGGGACACTG
CTGGGCTTTCAGCAGACCCCGAGACCTTTGCCAAGAACCGTGAACTCGAGGTGATCCACTGCCGCTGGGCCATGCTCGGTG
CACTGGGCTGTGTCTTCCCCGAAATCCTCTCCAAGAATGGTGTCAAGTTCGGTGAGGCAGTTTGGTTCAAGGCTGGAGCCC
AAATTTTCTCCGAAGGTGGCCTTGATTACCTTGGCAATCCAAACCTGATCCATGCCCAGAGCATCCTTGCAATCTGGGCTTGC
CAAGTAGTGCTCATGGGCTTCATTGAGGGCTACCGGGTTGGCGGAGGCCCACTTGGCGAAGGACTCGACAAAATCTACCCC
GGAGGAGCCTTTGACCCACTTGGACTTGCTGATGATCCAGAAGCATTTGCAGAGTTGAAGGTGAAGGAGATCAAGAATGGA
CGGCTGGCGATGTTTTCAATGTTTGGATTCTTCGTCCAGGCCATCGTTACTGGAAAGGGCCCAATTGAGAACCTCTTCGACCA
TCTCGCTGATCCTGTGGCCAACAATGCTTGGGCTTATGCCACTAACTTCGTACCTGGAAAATGA

>CSA035674.1
ATGTTGGGAGCCCTAGGTTGCATCACCCCAGAAGTCCTTGAAAAATGGGTCAAAGTGGACTTCAAAGAGCCTGTTTGGTTC
AAAGCTGGAGCCCAAATCTTCTCCAAAGGTGGCCTAGACTACTTGGGCAATCCAAATCTTGTCCATGCCCAAAGCATCCTGG
CAGTTTTGGGTTTCCAAGTGATTCTTATGGGCCTTGTTGAGGGCTTCCGCATTAACGGCCTTCCTGGAGTCGAAAAGGGAAA
CAACCTGTACCCCGGTGGACAGTACTTTGACCCACTGGGCCTTGCCGATGACCCGGTCACCTTTGCCGAGCTCAAGGTGAA
GGAAATCAAGAATGGCAGGCTAGCCATGTTCTCAATGTTTGGTTTCTTTGTCCAAGCAATTATCACTAATAAAGGCCCACTTG
AGAACCTGTTGGACCACCTTGACAATCCTGTGGCAAACAATGCTTGGGTCTATCATCTATGCCACCAAGTTTGTACCCGGGT
CATAAATTCATGA

>CSA019509.1
ATGTTGCGAGCCCTAGGTTGCATCACCCCAGAAGTCCTTGAAAAATGGGTCAAAATGGACTTCAAAAAGCCTGGTTCGTTC
AAATCTGGAGCCCAAATCTTCTCCGAAAGTGGCCTACACTACTTGGGCAACCCAAATCTTGTCCATACCCAAAGCATCCTGG
CAGTTTTAGGCTTCCAAGTGGTTCTAATGGGCCTCGTTGAGGGCTTCCACATTAACGGCCTTCCTAGAGTCGAAGAGGGAAA
CAACCTGTACCCTGGTGGACAATACTTTGACCCACTGGGCCTTGCCGATGACGCAGTCACCTTTGCCGAGCTCAAGGTGAA
AGAAATCAGGAATGGTAGGCTAGCCATGTTCTCAACGTTTGGGTTCTTTGTCCAAGCTATTGTCACTAGTAAAGGCCCACTT
GAGAACCTGTTAGACCACCTTGACAATCCTGTGGCTAACAACGCTTGGGTCTATGCCACCAAGCCGATCTTTGATCGGCGAG
CGGGGGTGCTCATAGATGACCTTCCCCAATCGTACCCAAGTTCCCGAGCTGAGGCAACTTTGGTGATGACGGACACGTGGC
CTTAG

>CSA002361.1
ATGGCTTCAATGTTAATGGCAGCTACTGCAAGCACTAGCACTGCCCTCAGACCAACCCCTTTTCTGGGTCAAACCAGGGGAC
CTAATGCCAACCCTCTTAGAGATGTTGTCTCTATGGGCACTGGCAAATTCACCATGGGAAATGAATTATGGTATGGACCGGAC
CGACTGAAGTACTTGGGACCCTTTTCAGCTCAGACTCCGTCGTACCTCACCGGAGAATTTCCCGGCGATTATGGATGGGACA
CTGCTGGTTTGTCTGCTGATCCTGAGGCCTTTGCCAGGAACAGAGCTCTTGAGAAAATTGAGAAACCTATGGGGGAAAAAA
ATGTAAAAGTCATCCACGGGCGATGGGCCATGCTCGGAGCCCTAGGCTGCATCACCCCAGAAGTCCTTCAGAAATGGGTAC
GGGTGGCCTTCAAAGAGCCAGTATGGTTCAAAGCCGGAGCCCAAATTTTCTCCGAAGGTGGCCTAGACTACTTGGGCAACC
CTAACCTAGTACATGCCCAAAGCATCCTCGCCGTGCTGGGCTTCCAAGTTGTTCTAATGGGCCTCGTTGAGGGCTTCCGCATC
AATGGCCTCCCCGGGGTAGGAGAGGGAAACAATCTCTACCCCGGTGGCCAATACTTTGACCCATTGGGTCTTGCCGATGACC
CGGTCACCTTTGCCGAGCTCAAAGTGAAGGAGATCAAGAATGGGAGGCTAGCTATGTTCTCAATGTTTGGGTTCTTTGTCCA
AGCTATTGTGACTGGGAAGGGCCCACTTGAGAACCTCTTGGACCACCTTGAAAACCCTGTGGCTAACAATGCTTGGGTCTAT
GCCACCAAGTTTGTGCCTGGGTCATAA

>CSA016997.1
ATGGCAACAATGTCAACTGCAACCAGCACTAGCACTGTCCTCAGACCCACTCCATTTCTGGGTCAAACCAGGGGAGCAAAT
GCCAACCCTCTCAGAGATGTTGTCTCTATGGGCATTGGCAAATTCACCATGGGGAATGAATTATGGTACGGGCCGGACCGGG
TGAAGTACTTGGGACCCTTTTCAGCTCAGACTCCGTCATATCTGACGGGAGAATTTCCCGGCGATTATGGATGGGACACTGCT
GGTTTGTCTGCTGATCCTGAAGCTTTTGCCAGGAACAGGGCTCTTGAGGTCATCCACGGAAGATGGGCCATGTTGGGAGCC

CTAGGTTGCATCACCCCAGAAGTCCTTGAAAAATGGGTCAAAGTGGACTTCAAAGAGCCTGTTTGGTTCAAAGCTGGAGCC
CAAATCTTCTCCGAAGGTGGCCTAGACTACTTGGGCAACCCAAATCTTGTCCATGCCCAAAGCATCCTGGCAGTTTTGGGTT
TCCAAGTGGTTCTTATGGGCCTTGTTGAGGGCTTCCGCATTAACGGCCTTCCTGGAGTCGGAGAGGGAAACAACCTGTACCC
CGGTGGACAGTACTTTGACCCACTGGGCCTTGCCGATGACCCGGTCACCTTTGCCGAGCTCAAGGTGAAGGAAATCAAGAA
TGGCAGGCTAGCCATGTTCTCAATGTTTGGGTTCTTTGTCCAAGCTATTGTCACTGGTAAAGGCCCACTTGAGAACCTGTTGG
ACCACCTTGACAATCCTGTGGCTAACAATGCTTGGGTCTATGCCACCAAGTTTGTACCCGGATCATAA

>CSA004532.1
ATGGCTTCACTGGCAGCATCAACGGCGGCTGCCTCCCTTGGCATGTCAGAAATGCTCGGAAACCCTCTCCGGAGTGGCGTA
ACGAGATCGGCACCTCCTCCCACCGCGACATCTAGCCCTGCCACCTTCAAGACCGTCGCACTTTTCTCCAAGAAGAAGGCT
GCACCTCCCAAAAAGGCTGTCGTCTCCCCCGTTGATGACGAGCTCGCCAAGTGGTACGGTCCCGACAGAAGAATTTTCTTG
CCGGAGGGGCTGTTGGACCGATCAGAAATTCCGGCATACCTCACCGGAGAAGTCCCTGGAGATTATGGTTACGATCCTTTTG
GGCTTAGCAAGAAACCAGATGACTTTGCCAAGTGGGCAATGTTGGGGGCTGCTGGCTTCATCATCCCTGAGGCCTTCAACA
AATTTGGTGCTAACTGTGGCCCTGAAGCTGTTTGGTTCAAGACAGGAGCTCTACTCCTAGATGGTAACACACTGAATTACTT
TGGAAAGAACATCCCCATTAATCTTATATTCGCTGTCATCGCTGAAGTTGTTCTTGTTGGTGGTGCTGAATACTACAGAATCAT
CAATGGATTGGTATGCGGTCCATTTGATCCATTGGGGCTTGCAAAGGATCCAGACCAGGCTGCAATACTGAAGGTGAAGGAG
ATCAAGAACGGTAGACTTGCAATGTTTGCAATGCTCGGTTTCTTCATCCAAGCTTATAATTTGGAGGACAAGCTTCACCCAGG
CGGTCCATTTGATCCATTGGGGCTTGCAAAGGATCCAGACCAGGCTGCAATACTGAAGGTGAAGGAGATCAAGAACGGTAG
ACTTGCAATGTTTGCAATGCTCGGTTTCTTCATCCAAGCTTATGTAACGGGAGAAGGTCCAGTTGAAAACCTCGCCAAACAT
CTAAGCGATCCGTTTGCAAACAACTTGCTCACTGTGCTTGCTGGATCTGCTGAAAGAGCTCCTACCCTGTGA

>CSA003567.1
ATGGCTATGCTTCAGCAATCTTCAATTTCGTCTAGCGGCCTCCTATCGAAAGGCGCAGCCCCTAAGTCACTCAATATTTATACA
AATTCCCAACCCCTCGCCAAACCTTATCGTGCCTGCAAAGCTTCATGGCAAGAGCTTGTGGGTGTCTTAATATTCTCGGCGGT
TCCTTTCACGGCGGTGAAAGCTATAGCCAACAGTCCCCTGGGAGAGTTGCTTCAGAGGCGATTGGAAGAGAAAAAGAAGG
ATGCCATCGATAATTCTTCCAATTTCAAGGCACTTGCTCAAATGGCTAGAAAGGATAGTTTATGGTATGGAGAGAAGCGTCCC
CGTTGGCTTGGTCCAATTTCATATGACTATCCTTCATATCTGACTGGAGAACTACCAGGGGACTATGGCTTTGATATTGCAGGT
TTAAGCAGGGATCCTGTGGCTTTCCAGAAATATTTCAACTTTGAAATACTGCATGCTCGCTGGGCCATGCTTGCAGCGCTTGG
TGCTCTGATTCCCGAACTATTAGACCTAGTAGGAGCCTTTCACTTTGTTGAGCCGGTCTGGTGGAAAGTTGGATATTCAAAGC
TTAAGGGTGACACATTGGACTACCTTGGCATCCCTGGGCTCCACTTAGCTGGAAGTCAAGGAGTGGTTGTTATAGCTATCTGC
CAAGCTCTTCTGATGGGGTTTGGTGTGACCATTGAAGGTCCTGACTGA

>CSA016587.1
ATGGCAACTCAAGCACTGGTGTCTTCATCATCTCTTACCTCCTCAGTGGAGGCTGCAAGGCAGATTCTAGGAGGAAGGCCAG
CTACCCATTCTTCAAGAAGGAAGGTCTCTTTTGTTGTTAGGGCAGCTACTACTCCCCCTGTTAAGCAAGGAGCAGATAGACC
TCTCTGGTTTGCCTCCAAGCAAAGTCTCACCTACTTAGATGGCAGCCTGCCCGGCGACTACGGATTCGACCCGCTCGGCTTG
TCCGACCCAGAAGGTACTGGAGGCTTCATCGAGCCCAGATGGCTAGCCTATGGCGAGATCTTCAACGGCCGTACCGCCATGG
TCGGCTCTATCGGATGCATCGCCCCAGAAATCTTGGGCAAACTCGGCCTAATTCCGCCAGAAACCGCTCTGCCGTGGTTCAA
AACAGGCGTGATCCCGCCCGCTGGGACCTACGACTATTGGGCTGACCCATACACTCTTTTTGTTTTCGAATTGGCACTAGTGG
GCTTTGCAGAGCACAGGAGGTTCCAGGCTTGGTACAACCCAGGCTCAATGGGTAAACAGTACTTTTTGGGCCTGGAGAAAT
ATTTGGGCGGGACGGATAACCCTGCATACCCTGGTGGGCCACTGTTTAACCCACTTGGGCTTGGAAAGGATGAGAAGTCAAT
GAGGGATATGAAGTTGAAGGAGGTAAAGAACGGGAGGTTGGCCATGTTGGGTATGTTGGGTTTCTTTGTGCAGGCGTTGGT
GACTGGGGTTGGACCCTTCCAGAACCTTCTGGATCATTTGGCTGACCCTGTCAACAACAATGTCTTGACCAACCTCAAGTTC
CACTAA

>CSA024064.1
ATGGCTTCCAAAGCCCTAATGAGCTGCGGCATCGCCGCCGTCTGCCCGTCAGTCCTTTCCTCTTCCAAGTCCAAATTTGCCGC
CGCGTTGCCGCTTCCAAGTGGTGGTGCCACCGCTACCTCCCGGCTCACCATGACGGCTGACTGGATGCCTGGCGAGCCAAG
GCCACCCTATCTTGACGGCTCCGCACCCGGTGATTTCGGGTTCGACCCGCTTCGTCTGGGTGAAGTCCCAGAAAACCTTGAA
AGATACAAGGAGTCTGAACTCATTCACTGCAGATGGGCTATGCTTGCTGTTCCAGGGATCCTAGTTCCAGAGGCCTTGGGAT
TGGGCAACTGGGTACAAGCTCAAGAGTGGGCGGCAATCCCTGGAGGACAAGCCACCTACCTTGGCCAACCTGTCCCATGGG
GCACCCTCCCAATCATCTTGGCCATTGAATTCCTTGCCATCTCCTTCGTCGAGCACCAGCGCAGCATGGAAAAGGACCCTGA
GAAGAAGAAGTACCCCGGTGGAGCTTTCGACCCATTGGGATACTCCAAAGACCCAGTCAAGTTTGAGGAGAACAAGGTCA
AAGAAGTAAAAAATGGCCGGCTTGCCTTGTTGGCTTTCGTGGGGATCTGTGTTCAACAGTCCGCTTACCCAGGGACAGGAC
CGTTGGAGAACCTGGCAACTCACTTGGCTGATCCATGGCACAACAACATTGGCGATATCATTATCCCTAGATCAATTTCTCCA
TGA

>CSA032834.1
ATGGCAACCATTACGGCACAAGCATCCACCGCTGTCTTCCGGCCACGTGCCGCCAAATCCCAGTTCCTTACCGGTTCTTCCG
GCAAGCTCAACAGAGAAATTTCACTTAAATCAAAATCTTCATCACCAAGATCATTCAAAGTTGAAGCCAAAGGTGAATGGTT
ACCGGGATTGCCCTCACCAGACTACCTTAATGGCAGTCTCCCTGGTGACAATGGATTTGATCCTCTAGGCCTTGCAGAGGAC
CCTGAGAACCTAAAATGGTACATCCAAGCCGAGCTTGTGAACAGCCGGTGGGCCATGTTAGGGGTCACCGGAATGCTGCTG
CCAGAAGTGTTCTCAACTATCGGAATAATCAATGTCCCCAAATGGTACGATGCAGGAAAAGCCGAGTACTTCGCGTCATCAT
CAACTCTGTTCGTGATCGAGTTCATCTTGTTCCACTACGTTGAGATCCGACGATGGCAGGACATCAAGAACCCAGGAAGTGT

CAACCAAGATCCTATCTTCAAGAACTATAGCTTGCCTCCCGGTGAGGTTGGGTACCCTGGTGGCATTTTCAATCCCCTGAATT
TTGCACCCACCCAGGAGGCCAAGGAGAAGGAGCTTGCCAACGGGAGATTGGCAATGTTGGCATTCTTGGGATTCATCGTTC
AGCACAATGTGACCGGAAAAGGGCCATTCGACAACCTCTTGCAGCATCTCTCCGACCCATGGCACAACACAATTATTCAGAC
ATTCCAGGGTTACTAA

>CSA013374.1
ATGGCTCAGACCATGTTGCTCACTTCTAGTTCAAGTGTCAATACTCATGCATTGGATTTGAAGAGACAACCATTACTTGAAAG
TCTAAGGCCCAAACCATTCTCTCACATCTTACTACCTCCACTTCCATCTTCTTCTTCATTTCATCAGACTACCACCATTGCTCTC
TTCAAATCCAAAACCAAAGCTGCCCCTGTCAAGAAGGTTGCCGAGCCTAAGCCGAAGGTTGAAAGTGGTATTTTTGGCACG
TCGGGTGGGATTGGGTTTACAAAGCAAAATGAGCTTTTTGTGGGTCGTGTTGCCATGATCGGCTTCGCTGCATCTTTGTTGGG
AGAAGCAATAACAGGGAAAGGAATCCTAGCACAACTGAATCTGGAGACTGGAATTCCCATCTATGAAGCTGAGCCTCTTCTC
CTCTTCTTCATCCTTTTCACCCTCCTCGGAGCCATCGGAGCTTTGGGCGATAGGGGTCGCTTTGTTGATGACCCCCCTACCGG
GCTTGACAAGGCTGTCATCCCTCCAGGCAAAAGTCTCAGATCAGCATTGGGTCTTAAAGAAGGAGGTCCACTATTTGGATTC
ACAAAGTCGAATGAGCTGTTCGTGGGACGATTGGCTCAGTTGGGAATTGCATTCTCTATAATTGGAGAGATAATCACGGGGA
AAGGAGCTCTGGCACAGCTAAACATCGAGACAGGAGTTCCAATCAGCGATATTGAGCCCCTTGTGTTGTTCAATGTCCTCTT
CTTCTTTGTCGCCGCATTGAATCCTGGAACTGGCAAGTTTCAAACAGATGAAGAAGAGTAG

>CSA011328.1
ATGTACTCACCTATCCATCAAGATAAGCCTCTTGAAAATCCATACATTAAACAGCCTCTCATATCTCTCATTCACACAAACCAA
TCAAACACTCAAAATTCATCAAAAGAAAATCAGCCAAACATGGCCTCCTCTGCTTTTGCTTCTTCAGCTATTGCAGCTGTTG
GCCTCTCTTCACCCAGTTCCAAAAAGAGTGGATCAATTGTGGGAGCAACAAAGGCTTCATTCTTTGGAGGGAGAAAACTAA
GACTGAGAAAGTACAGTACATCACCGGCCGGAGCACGGTCGGTTACCGTTTGCGTCGCAGCCGATCCCGATAGGCCCATCT
GGTTCCCAGGCAGCGTCCCTCCTCCATGGCTTGATGGAAGCCTCCCTGGAGACTTCGGATTTGATCCCCTCGGTCTTGGATCT
GACCCTGAGACTCTGAGATGGAACGTCCAATCCGAGCTAGTCCACTGCAGATGGGCAATGTTAGGCGCTGCTGGAATCTTCA
TCCCTGAATTCCTTACAAAGATTGGAATCCTCAACACCCCATCATGGTACACCGCGGGTGAGCTCGAATACTTCACAGACAC
CACCACTCTTTTCATCATCGAGCTTATTTTAATTGGTTGGGCCGAAGGAAGGCGTTGGGCTGACATACTCAAGCCAGGTTGTG
TCAACACCGATCCGATTTTCCCCAACAACAAGCTCACCGGCACAGATGTTGGGTACCCGGGTGGGCTGTGGTTTGACCCATT
GGGTTGGGGAAGCGGGTCACCAGAGAAAGTGAAGGAGTTGAGGACTAAGGAAATTAAGAACGGAAGGCTGGCTATGTTGG
CTGTGATGGGTGCATGGTTTCAAGCTATTTATACAGGGACTGGACCTATTGACAACCTCTTTGCCCACCTTGCTGATCCTGGT
CATGCCACCATTTTTGCTGTAAGTGCATTTTAA

>CSA035688.1
ATGGCTGCAACATCAGCTCCAGTGGGCTCTCCCTTCTTGTCCGGTGGGAAAAGAAGCCAGACGTTGTTGTTGTCTGCAACCA
TTGGAGCTAGATCACTTGGTGGCGGTGTGGTGGCTCCTAAGAAGTTTGTTCTGCTGGCTGCTGCTCTTAAGAAGTCTTGGAT
CCCTGCTGTCAAAGGTGGTGGCAGCTTCATCAACCCCGAATGGCTCGATGACTCGCTACCAGGGGACTATGGTTTTGACCCT
CTGGGCCTGGGAAAGGACCCTGCATTCCTCAAGTGGTACAGAGAGGCCGAGCTCATCCATGGCCGGTGGGCAATGGCTGCG
GTGGTCGGCATCTTTGTTGGGCAAGCCTGGAGCGGAATCCCTTGGTTTGAAGCCGGTGCTGACCCCAGCGCCATTGCTCCCT
TCTCCTTCGGCACCCTCCTCGGCACCCAACTCCTCCTCATGGGATGGGTAGAGAGCAAGAGATGGGTTGATTTCTTCAACCC
AGAGTCGCAGTCTGTTGAGTGGGCCACCCCATGGTCGAGAACGGCGGAGAATTTTGCCAATGCCACCGGTGACCAGGGATA
CCCAGGCGGCAAATTCTTTGACCCATTGTGCCTGGCCGGGACAATCGTCAACGGAGTATACGTTCCGGACACAGAGAAGCT
GGAGAGGCTGAAGCTGGCTGAGATTAAGCATGCCAGGATTGCTATGTCGGCTATGTTGATTTTTATTTTGAGGCTGGACAAG
GGAAGACACCTCTAGGAGCTCTTGGTTTGTAA

>CSA016010.1
ATGGCCACGTCAGCAATGCAGTCCATCCTGGCAAGACCAGTGACAGGTGTCACGACGAGAGCAAGGTTCAGTCAGTCCACT
CCTTGCAGCTACGTGCCACGTCTGCGGAGGAATGCTGGCATGCAAGTGCGGTGCATGGCAGAGGATGGTCAAAATAAGGAT
CCGACACCCACTACAACTCCTCCGCCGGCATCACAGCCAATCCCTTCTCCTCCACCAAAGCCAAAGGTAAGCACCAAATTTT
CTGACGTGTTAGCATTCAGTGGACCCGCACCGGAGAGAATCAACGGCAGGCTGGCGATGATCGGGTTCGTGGCCGCGATGG
CGGTGGAACTATCGAAGGGTGAGGATGTGTTGGCCCAGATATCCAATGGTGGAGTTTCATGGTTTGTGGGGACAAGCATTGT
GCTAACCCTAGCATCTTTGATACCTCTATTCAAAGGGGTGAGTGTGGAGTCTCGTTCAGAGGGGATTATGAGCTCTGATGCTG
AGCTCTGGAATGGGAGGTTTGCTATGTTGGGTTTGGTTGCATTGGCTTTCACTGAGTTTGTCAAAGGTGGAGCCCTTGTGTA
G

>CSA016009.1
ATGGCCACGTCAGCAATCATGCAGTCCGTCCTGGCAAGACCGGTGACAAGTGTCACAACGAGGGCTAGGTTTGGTCAGTTT
ACTCCTTGCAGCTACGTGCCATATCTGCAGAAGAATGCTAGCATGCAAGTGCGGTGCATGGCAAAGTATGGTCAAAAAGAG
GACGTGACACCGAAGCCAAAGGTAAGCACCAAATTCTCTGACGTGTTAGCATTCAGTGGACCCGCACCGGAGAGAATCAAC
GGCAGGCTGGCGATGATAGGGTTCGTGGCGGCGATGGCGGTGGAGCTATCGAACGGCGAGGATGTGTTGGTCCAGATATCC
AACGGTGGAGTTCCATGGTTTGTGGGGACAAGCATTGTGCTAACCCTAGCATCTTTGGTACCTCTATTCAAAGGGGTGAGTG
TGGAGTCTCGTTCAGAGAGGATTATGAGCTCTGATGCTGAGCTCTGGAATGGGAGGTTTGCTATGCTGGGTTTGGTTGCATT
GGCTTTCACTGAGTTTGTCAAAGGTGGAGCCCTTGTGTAG

>CSA008855.1
ATGAACTTCCAGTTCAACCACCGAGAGTGGTGGTCCAGTGGTGAAGCCCTCGCGCTGTACGCCGATCGACATTTGGCTATGA

TTGGGGCAGGTGAATCCGGACCACCGGGCCTGGGCCTTCGGAGGCTTCCAGGTGACTACGGATTTGACCCACTAGGCCTAG
GAAAGGACCCAGCATTCCTCAAATGGTACAGAGAGGCTGAGCTAATCCACGGCCGATGGGCGATGGCGGCAGTGGTTGGCA
TCTTCGTTGGGCAAGCCTGGAGCGGCATCCCGTGGTTCGAAGCAGGTGCCGACCCAGGCGCCATCGCCCCTTTCTCCTTCG
GCACCCTCCTCGGCACCCAACTCCTCCTCATGGGATGGGTTGAGAGCAAGAGATGGGTTGACTTTTTCAACCCAGAATCCCA
GTCGGTTGAGTGGGCCACACCGTGGTCGAAGACCGCGGAGAATTTTGCCAACGCGACCGGTGACCAGGGCTACCCGGGTG
GAAAATTCTTCGACCCGTTGTGCCTGGCTGGCACGATCGTCAACGGGGTGTACATTCCAGATAAGGAGAAGTTGGACAGAC
TGAAATTGGCCGAGATTAAGCATGCTAGGCTTGCTATGGTGGCTATGTTGATTTTCTACTTTGAGGCTGGACAAGGGAAGAC
ACCCCTTGGAGCTCTTGGATTGTAA

>CSA011380.1
ATGGCCTCATCTGTTTGTGCTTCTTCAGCTTTTGCAGCTGTTGGCCTCTCTTCCCCAAGTTCCCAGAAGAGTGGATCAATTGT
GGGAGCAACAAAGGCTTCTTTCCCAGGAGGGAGGAAACTGAGACTCAGAAAGAATGCAGCACCGGCTGGAACACCATCA
GGATCGGTTACCGTCTGTGTCGCAGCGGATCCTCATAGGCCACTCTGGTTCCCCGGCAACACCCCTCCTCCCTGGCNATTAG
TGAAGTGGGATAAACATACAGACAATGCACAATGCTTGTCACATAAGAGTGGATCAATTGTGGGAGCAGCAAAGGCTTCTTT
TCTAGGAGGGAGGAAACTGAGACTGAGAAAGAATGCAGCACCGGCTGGAACACCATCAGGATCGGTTACCGTCTGTGTCG
CAGTGGATCCTGATAGGCCACTCTGGTTCCCCGGCAGCACCCCTCCTCCCTGGCTTGACGGCAGTTGTTATGTTACTGAAGTT
GTAGCTGAACCTTGTGATGCAGCCTCCCTGGAGACTTTGGATTTGATCCACTTGGTCTTGATCTCTAAAAGCTTTGAGCCACT
ACTAACAGGATCTGATCCAGAGACATTGAGATGGAATGTACAAGCAGAGCTAGTGCACTGCAGATGGGCAATGCTGGGCGC
TGCTGGCATCTTCATCCCAGAATTCCTCACAAAGCTCGGCATTCTAAACACCCCTTCATGGTACACTGCTGGCGAGCAAGAG
TACTTCACTGACACCACCACTCTCTTCATCGTCGAGCTCATTTTCATTGGCTGGGCGGAGGGAAGGCGGTGGGCTGACATCC
TCAAGCCGGGCTGTGTAAACACCGACCCCATCTTCCCCAACAACAAGCTTACAGGTACTGATGTTGGATACCCGGGTGGGCT
ATGGTTTGACCCACTCGGGTGGGGAAGCAGTTCACCGGAAAAGATCAGGGAGTTGAGGACAAAAGAGATCAAGAATGGGA
GGTTGGCTATGTTGGATGTGATGGGTGCTTGGTTCCAAGCCATTTACACAGGGACTGGACCTATTGACAACCTCTTTGCACAC
CTTGCTGATCCTGGCCTTCACCCCCAAGTGATAGGAGCAAGTGAACAAGCATGCTAA

>CSA014653.1
ATGGCAGCCACCACGTCCGCCGCCGCCACCTCATCATTCATCGGAACACGGTTCCCCGAAGTCCACTCCGGTTCGGGGCGA
GTCCAAGCCCGGTTCGGATTCGGGGCCAAGAAGGCTCCGGCGAAGAAATTTGCGAAGCCGGGTTCGGACCGTCCGCTGTG
GTTCCCGGGAGCAAAAGCACCCGAGTGGTTGGACGGAACATTGGTGGGAGATTACGGGTTCGACCCGTTCGGGTTGGGGA
AACCGGCGGAGTACTTGCAATTCGAGTTGGACTCGTTGGATCAAAACTTGGCGAAGAACTTGGCGGGTGATATAATCGGGA
CCCGATTCGAAAGCGCGGATGTAAAATCGACACCGTTTCAGCCCTACAGTGAGGTGTTTGGGTTGCAGAGGTTTAGGGAGT
GTGAGTTGATTCATGGAAGGTGGGCCATGTTGGCTACGCTGGGTGCACTTACGGTGGAGTGGCTCACTGGCATTACGTGGCA
AGATGCCGGAAAGGTGGAGCTACTTGAAGGGTCATCCTACCTTGGCCAACCACTTCCATTCTCCATAACCACATTGATATGGA
TTGAGGTCCTAGTCATTGGCTACATTGAGTTCCAGAGGAATTCCGAACTCGACCCGGAGAAGAGGCTGTACCCAGGGGGTA
AATACTTCGACCCTCTAGGTTTAGCCTCTGACCCTGAGAAGAAGGCAACCCTCCAACTTGCTGAGATCAAGCACGCCCGCCT
TGCCATGATTGCCTTCCTTGGGTTCGCTGTCCAAGCGGCTGCCACTGGCAAAGGCCCGCTCAATAACTGGGCGACCCATTTG
AGTGACCCGCTTCACACCACCATTATTGACACCTTTTTCTCTTGA

>CSA020482.1
ATGGCCCTTTCTATTGCTTCTACTGCACTCTCAAGTCTCCCAATAAGGAAAATACCTGGAAAAGCTCCAGGAAAGATTGCTAC
AGGTTTAGCTTGGAAAACTAGTGTGAATGCAACAAAAGGAGGGGTGTCAAGTGTTTGTGAACCACTTCCTCCTGATAGGCC
ATTGTGGTTCCCTGGTAGTTCACCTCCCGAGTGGCTTGACGGCAGTCTTCCTGGAGATTTTGGCTTTGACCCACTTGGATTAG
GGTCTGACCCGGAGTTACTCAAATGGTTCGCACAAGCAGAGCTAATGCACGCTAGATGGGCAATGCTGGCTGTCGCGGGGA
TTCTCATTCCAGAATGGCTTGAAAGCCTCGAATTAATTGAGAACTTTTCATGGTATGATGCCGGTGCTAGAGAATACTTTGCA
GACCCAACAACCCTATTTGTGGTGCAACTTGTCTTGATGGGCTGGGTAGAAGGCCGGAGATGGGCCGATATGGTCAACCCGG
GTTGCGTTGACATCGAGCCTACCTATCCAACTAAGAAAAAACCGAAGGCAGATGTTGGGTACCCGGGGGGGTTATGGTTTGA
TCCGTTTATGTGGGGGAGAGGATCGCCTGAGCCAGTGATGGTGTTGAGGACTAAAGAGATCAAGAATGGGAGGCTTGCAAT
GCTAGCTTTTGTGGGCTTTTGTTTCCAAGCCATTTATACTGGACAAGGCCCCATTGAGAACTTGATGGCTCACATTGCTGATC
CTGGCCACTGCAACATCTTTTCGGTACGTCGCCTTTGA

>CSA010862.1
ATGGCCGCAACCACCGCCGCCGCCGCTGCCGCCACATCATCATTTCTAGGCACCCGCCTCGCCGACCTATGTTCCGGTTCGG
GCCGGGTCCAGGCCCGGTTCGGATTCGGACGCAAAAAGGCTCCACCAAAGAAGATTGCGAAGCAGGGCTTTGACCGCCCA
CTTTGGTTCCCGGGAGCGAAAGCGCCCGAATGGTTAGATGGGAGTCTTGTCGGGGATTACGGGTTTGACCCGTTCGGGTTG
GGTAAACCGGCTGAGTACTTGCAATTTGATTTGGACTCGTTGGATCAGAACTTGGCTAAGAACTCGGCGGGTGATGTAATCG
GGACCCGGTTCGAGAGCGCGGATGTGAAGTCGACGCCGTTTCAGCCGTACACAGAGGTGTTTGGGTTGCAGAGGTTTAGGG
AGTGTGAGCTGATTCATGGAAGGTGGGCTATGTTGGCTACGCTCGGCGCGCTTACTGTTGAGTGGCTCACTGGTGTTACGTG
GCAAGACGCTGGAAAGGTGGAGCTAATTGAAGGGTCATCCTACCTTGGCCAACCACTTCCATTTTCCATAACCACATTGATAT
GGATTGAGGTCATAGTCATTGGATACATAGAGTTCCAAAGGAATGCTGAGCTTGACCCAGAAAAGAGGCTCTACCCGGGTG
GAAAATTCTTCGACCCGCTTGGCTTGGCCTCAGACCCAGAGAAGAAGGCAACCCTCCAATTGGCGGAGATCAAGCATGCCC
GCCTTGCCATGGTAGCCTTCCTAGGTTTTGCCGTCCAAGCTGCTGTCACCGGCAAAGGGCCACTCAACAACTGGGCGACCC
ATTTGAGTGACCCGCTCCACACAACCATTATAGACACCTTTTTCTCTTGA

>CSA012994.1

CTACCTGGTGACAATGGGTTCGATCCCTTGGGGCTAGCCGAGGACCCAGAGAACTTGAAATGGTTCATCCAGGCCGAGCTT
GTGAACAGTCGGTGGGCCATGTTGGGTGTCACCGGAATGCTACTGCCGGAAGTGTTGAGCAGTATCGGAATAATCAACGTTC
CAAAATGGTACGATGCAGGAAAATCCGAATACTTTGCATCATCATCGACACTTTTCGTGATCGAGTTCATCTTGTTTCACTAC
GTGGAGATCAGACGGTGGCAAGACATCAAGAACCCTGGAAGTGTCAACCAAGATCCTATCTTCAAGAGCTATAGCTTGCCT
CCCAATGAGTGTGGCTACCCTGGTGGCATTTTTAACCCCCTCAACTTTGCTCCCACTGAGGAGGCCAAAGAGAAGGAGCTC
GCTAATGGTAAGTTCTTGAAATATCACGTGACCTTACTTTAA

5) Table S5. Primer pairs for cloning CsCP1 or CsCP2 and the qRT PCR of the *CsCAB* family

(1) the sequences of the primers for *CsCP1/2* gene cloning:

① The primer sequences for cloning comp56954_c0_seq1

CAB-F: CCGTGAACTCGAGGTGATCCACT

CAB-R: CCATTCTTGATCTCCTTCACCTTCA

② The primers for *CsCP1/2* gene RACE:

CsCP1f: GCTGCTGGCTTCATCATCCCTGA （The primer was used to clone the 3'-terminal sequence of CsCP1 gene in combination with our library universal downstream-primer.）

CsCP1s: GCAGAGCAATGGCTTCACTGGCA

CsCP1x: GTACTCAGATCAAAGGCACATGCAT

CP1q1: GTGCGGGCGGTGAAGGCACA

CP1qf: GCACCTCCCAAAAAGGCTGTCGTCT

CP1qd: CTTGGCGAAGTCATCTGGTTTCTTGCT

CPIq2: CTGACGGGGTTAATGCAGGAGTGTCA

CsCP2f：GGAAAGGGCCCAATTGAGAACCTCT (The primer was used to clone the 3'-terminal sequence of CsCP2 gene in combination with our library universal downstream-primer.）

CP2r：CCACCTCCAAAGCTGCCAATCT (The primer was used to clone the 3'-terminal sequence of CsCP2 gene in combination with our library universal upstream-primer.）

CsCP2x: CAGCTCAATCTTCATACTTCATTTTCCAGGT

CsCP2-R1：CCGAGCTCTTCATTTTCCAGGTACGAAGTTAGTGGCA

CP2qf: CAGTCGGCTTTCGCTGGACAGACT

CP2qd：GGAATTCACCGGTCAAGTATGATGGAGT

(2) The primers sequences used for ten gene qRT PCR in the *CsCAB* family, which were designed in their differential sequence regions:

① Q030474F：GGAAGGATCAGCATGAGGAAGACTGGT
Q030474R：CGGCCAAAAGCTCGGGGAAGACA

② Q030476F：GGAAGTCTTTTGGCAAGCCAGGCCCATCAGGT
Q030476R：CCGTTGCGGGCCAAGAGCTCT

③ Q008917F：TCTACCATGGCTCTCTCTTCCCCATCT
Q008917R：GAGGTAAGATGGGGGATCACCAGACA

④Q035910F：CACTGGGCAATTCCCTGGTGATTATGGT
Q035910R：CAACAGCACCCATCAAGATAACTTGGCAAG

⑤ Q019572F：CGCATCACCATGAGGCGCACTGTGA
Q019572R：CATTCTTGGAGAGGATTTCGGGGAAGACA

⑥ Q002361F：AGGGGACCTAATGCCAACCCTCTTAG
Q002361R：CTGGCAAAGGCCTCAGGATCAGCA

⑦ Q016997F：CTGCAACCAGCACTAGCACTGTCCTCA
Q016997R：GCCGGGAAATTCTCCCGTCAGATATGA

⑧ Q004532F: GAGGGGCTGTTGGACCGATCAGA
Q004532R: GAACCAAACAGCTTCAGGGCCACAGT

⑨ Q016587F：GGAAGGTCTCTTTTGTTGTTAGGGCAGCT

Q016587R：CCATAGGCTAGCCATCTGGGCTCGA

⑩ Q024064F：CGTTGCCGCTTCCAAGTGGTGGT

Q024064R：GCCTCTGGAACTAGGATCCCTGGA

Actin-F：GCTCCTGAAGAGCATCCAGTGCT

Actin-R：GGCACAGTGTGGCTCACACCA

6) Table S6. The data table of the expression changes of 10 CAB genes in tea plant under different treatment conditions

Gene ID	Treatment Times	Wound treatment	Cold treatment	Mannitol treatment	NaCl treatment	ABA treatment	MeJA treatment
CSA030474	0 h	0.998	1.000	0.998	0.998	1.000	1.002
	3 h	0.456	1.079	0.010	0.132	0.261	1.530
	6 h	0.135	0.883	0.010	0.065	0.084	0.046
	12 h	0.034	0.274	0.290	0.010	0.970	0.126
	24 h	1.584	0.796	1.388	2.639	5.266	0.623
	36 h	0.018	0.796	0.107	0.017	0.009	0.059
CSA030476	0 h	1.000	1.000	1.000	1.000	1.005	0.998
	3 h	1.526	1.301	3.904	3.387	1.713	335.461
	6 h	0.937	1.741	2.141	1.072	1.733	3516.684
	12 h	0.256	1.133	0.145	0.318	6.528	0.328
	24 h	1.414	1.102	1.125	2.639	5.086	1.023
	36 h	0.060	1.376	0.084	0.271	0.274	0.186
CSA008917	0 h	1.002	1.000	0.998	1.005	1.002	1.000
	3 h	0.469	0.889	27.538	0.532	0.490	1.523
	6 h	0.405	0.768	26.173	0.058	0.219	7.656
	12 h	0.047	0.257	0.161	0.283	4.846	0.204
	24 h	0.556	0.785	0.500	0.000	0.910	0.606
	36 h	0.034	0.547	0.123	0.398	0.111	0.087
CSA035910	0 h	0.995	1.000	1.000	1.002	0.998	1.002
	3 h	0.329	0.824	0.048	0.388	0.582	13.674
	6 h	0.182	0.847	0.016	0.121	0.208	35.671
	12 h	0.066	0.212	0.072	0.053	3.031	0.181
	24 h	0.426	0.624	0.480	0.574	0.867	0.560
	36 h	0.030	0.727	0.061	0.176	0.103	0.070
CSA019572	0 h	0.998	1.000	0.995	1.002	1.000	1.000
	3 h	0.470	1.206	0.128	0.837	1.326	0.694
	6 h	0.229	1.464	0.013	0.168	0.435	0.705
	12 h	0.085	1.035	0.249	0.069	0.219	0.125
	24 h	0.538	1.548	1.478	0.000	2.019	0.961
	36 h	0.039	1.028	0.119	0.435	0.079	0.072

CSA002361	0 h	1.002	1.000	0.998	1.005	1.005	1.005
	3 h	0.120	1.659	0.014	0.712	0.413	1.069
	6 h	0.324	3.364	0.120	0.192	0.079	0.061
	12 h	0.104	1.840	0.137	0.031	0.073	0.064
	24 h	2.915	1.404	3.167	2.591	2.189	0.046
	36 h	0.207	0.966	0.099	0.076	0.024	1.488
CSA016997	0 h	1.000	1.000	0.998	1.000	1.003	1.000
	3 h	1.245	4.112	5367.371	2.153	0.688	325.535
	6 h	0.686	21.259	18011.949	0.140	1.464	2515.565
	12 h	1.002	210.839	0.292	0.016	139.102	0.217
	24 h	1.709	0.651	0.554	4.189	0.588	4.959
	36 h	12.817	1.414	0.408	0.058	2.417	0.183
CSA004532	0 h	1.000	1.000	1.000	1.005	1.002	1.002
	3 h	0.291	0.476	0.064	0.349	0.749	0.792
	6 h	0.374	0.555	0.055	0.191	0.456	1.149
	12 h	0.228	0.366	0.336	0.163	0.237	0.506
	24 h	0.365	0.514	0.310	0.000	0.606	0.418
	36 h	0.175	0.511	0.287	0.331	0.356	0.268
CSA016587	0 h	1.000	1.000	1.000	1.002	1.002	0.998
	3 h	0.166	0.655	0.017	0.085	0.462	1.505
	6 h	0.083	0.480	0.036	0.033	0.234	0.172
	12 h	0.114	218.275	0.344	0.000	0.130	0.202
	24 h	0.265	0.363	0.518	0.210	0.586	0.633
	36 h	0.023	0.366	0.175	0.049	0.237	0.157
CSA024064	0 h	1.002	1.000	1.002	1.002	0.998	0.998
	3 h	0.277	0.633	0.059	0.626	0.680	0.217
	6 h	0.214	0.774	0.170	0.195	0.235	1.014
	12 h	0.155	0.511	0.288	0.256	0.095	0.244
	24 h	0.254	0.511	0.415	0.380	0.739	0.407
	36 h	0.098	0.511	0.057	0.221	0.247	0.089

7) **Table S7. Comparison of sequence similarity between CsCP1 or CsCP2 and other homologous proteins in the same subfamily**

	The proteins coded by CsCP1/ CsCP2 homologue genes	Identities (length & percent)	Positives (length & percent)	Gaps (length & percent)
CsCP1	CSA030476	116/250(46%)	137/250(54%)	58/250(23%)
	CSA030474	114/250(46%)	136/250(54%)	58/250(23%)

	CSA016997	117/279(42%)	143/279(51%)	66/279(23%)
	CSA035910	108/259(42%)	128/259(49%)	76/259(29%)
	CSA008917	107/259(41%)	129/259(49%)	76/259(29%)
	CSA019572/ CsCP2	107/259(41%)	127/259(49%)	76/259(29%)
	CSA016587	91/239(38%)	117/239(48%)	50/239(20%)
	CSA002361	116/316(37%)	143/316(45%)	97/316(30%)
	CSA024064	66/239(28%)	97/239(40%)	73/239(30%)
CsCP2	CSA035910	197/230(86%)	211/230(91%)	1/230(0%)
	CSA008917	211/265(80%)	232/265(87%)	8/265(3%)
	CSA030476	210/268(78%)	230/268(85%)	10/268(3%)
	CSA030474	211/270(78%)	229/270(84%)	14/270(5%)
	CSA016997	188/264(71%)	207/264(78%)	12/264(4%)
	CSA002361	188/279(67%)	210/279(75%)	24/279(8%)
	CSA016587	104/247(42%)	138/247(55%)	38/247(15%)
	CSA024064	82/193(42%)	103/193(53%)	14/193(7%)
